# Immunomodulatory biomaterials for osteoarthritis: Targeting inflammation and enhancing cartilage regeneration

**DOI:** 10.1016/j.mtbio.2025.102100

**Published:** 2025-07-16

**Authors:** Ruizhe Zhao, Bing Liang, Yijie Shi, Jianfei Gao, Xuezhe Wang, Tianyi Shao, Kunyue Xing, Mingzhe Yan, Tianrui Wang, Yingze Zhang, Dongming Xing

**Affiliations:** a Cancer Institute, The Affiliated Hospital of Qingdao University, Qingdao University, Qingdao, Shandong, 266000, China; b Qingdao College of Medicine, Qingdao University, Qingdao, 266003, China; c UCL Institute of Health Informatics, University College London, Gower Street, London, WC1E 6BT, UK; d Department of Orthopedics, Affiliated Hospital of Qingdao University, Qingdao, 266000, China; e Orthopaedic Center, The Affiliated Hospital of Qingdao University, Qingdao, Shandong, 266000, China; f School of Life Sciences, Tsinghua University, Beijing, 100084, China

**Keywords:** Immunomodulatory biomaterials, Osteoarthritis, Cartilage regeneration, Immune response, Responsive biomaterials, Drug delivery

## Abstract

Osteoarthritis (OA) is a prevalent joint disorder characterized by progressive cartilage degradation, impaired mesenchymal stem cell (MSC) function, and chronic inflammation, ultimately leading to irreversible structural damage and functional impairment. Despite its high global burden, no regulatory agency has yet approved a disease-modifying therapy for OA, and effective interventions to halt or delay its progression remain a major challenge. Recent research highlights the pivotal role of the immune system in OA pathogenesis, with immunomodulatory biomaterials emerging as a promising strategy to simultaneously regulate inflammatory responses and promote tissue regeneration. These biomaterials, by leveraging their biocompatibility and immunoregulatory properties, offer a transformative alternative to conventional OA therapies, which predominantly focus on symptom management rather than targeting the underlying disease mechanisms. In this review, we comprehensively examine various immunomodulatory biomaterial strategies designed to mitigate OA progression. We first elucidate the immune landscape of OA, detailing the interplay between inflammation and disease pathophysiology. Next, we explore the latest advancements in immunomodulatory biomaterials, including nanoparticles (NPs), hydrogels, and scaffolds, highlighting their potential to reshape OA treatment. Finally, we discuss existing challenges and propose future directions for optimizing biomaterial-based immunotherapies to enhance OA management.

## Graphical abstract

**Figure:**
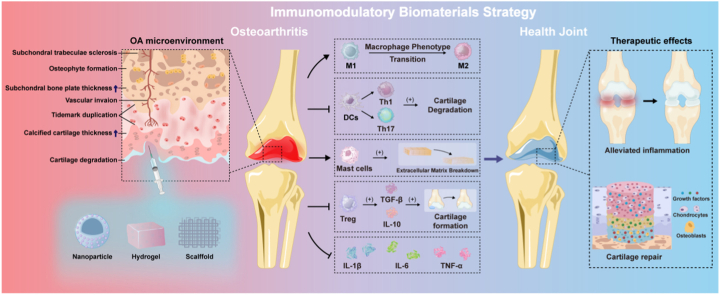

## 1 Introduction

Osteoarthritis (OA) is a progressive degenerative joint disease that affects not only the articular cartilage but also the entire joint structure, including subchondral bone, synovium, joint capsule, and periarticular muscles [1]. Its epidemiological tapestry is one of intricacy and multifaceted dimensions, wherein genetics, biology, and biomechanics find their symbiotic interplay [2]. With the global aging population, OA prevalence has surged to over 200 million cases, imposing a substantial socioeconomic burden and severely impairing patients' quality of life [3]. However, OA's complex pathophysiology hinders curative treatments. Key challenges include poor tissue regeneration, chronic inflammation, and unknown molecular drivers [3,4].

Current OA therapy includes both non-surgical and surgical approaches [5]. Intra-articular (IA) injections remain a central component of the non-surgical treatment modalities for OA, as IA injections provide brief pain relief, improve joint function and have a low risk of injury [6]. Surgical procedures, particularly joint arthroplasty, serve as the ultimate recourse for end-stage OA [7]. Conventional pharmacological treatments, including analgesics, corticosteroids, and physical therapy, aim to mitigate symptoms but fail to arrest disease progression [8]. Given these limitations, there is a critical need for the clinical development of innovative medications for the treatment of OA [9].

With the advent of biomaterials research, a cornucopia of biomaterial variants has burgeoned, unfurling a panoply of prospects for OA mitigation [10]. These multifarious biomaterial iterations, in the pursuit of remedying OA's affliction, have reaped gratifying outcomes [11]. Their prowess lies in the augmentation of pharmacological precision, the elongation of therapeutic duration, and the fine calibration of intervention [12,13]. But a frequent problem with implanting pure biomaterials is that they trigger undesirable immune reactions, which can lead to hyperinflammation, pain, tissue damage, fiber encasement, and even graft collapse [14]. Moreover, previous biomaterial-based OA therapy have predominantly focused on promoting osteogenesis while largely overlooking the pivotal role of immune regulation [15]. Given that inflammation serves as a fundamental driver of OA pathogenesis, integrating immunomodulatory strategies into biomaterial design is imperative for achieving sustained therapeutic outcomes.

*Arron* et al. first proposed the concept of "bone immunity" when studying the process of T cells activating bone resorption [16]. With the development of bone immunity, more and more studies have proved that bone regeneration is not a simple process of bone formation and absorption, but involves multiple systems, including the skeleton and the immune system [17]. Under physiological conditions, the immune system may protect bones, and the disruption of immune function may promote bone loss [18]. A profusion of investigations, underscoring the interplay of immunity in the pathogenesis of OA, delineates an enduring diorama of the chronic inflammatory tableau [15]. Immunomodulated inflammatory events directly drive synovitis and joint tissue destruction, creating conditions ideal for immunotherapies [19]. Key strategies demonstrating this potential include: selectively modulating macrophage polarization to limit OA progression, precisely targeting cytokines in the nuclear factor kappa-B (NF-κB) pathway, and regulating the complement system [20]. Concomitantly, the convoluted choreography enacted by the likes of interleukin (IL)-1β, IL-6, tumor necrosis factor-α (TNF-α), IL-10, IL-4, and IL-17 within the pathophysiological opera of OA, emerges as a theme meticulously woven into the saga [15]. The proposal and improvement of the principle of bone immunity provide a theoretical basis for subsequent strategies that combine biomaterials with immune mechanisms [21].

Biomaterials, including NPs, hydrogels, and scaffolds, have been extensively utilized for bone tissue repair and regeneration. These materials play distinct roles in the immunomodulatory treatment of OA due to their unique characteristics and the potential for modification through various physical or chemical methods. Most biomaterials investigated for OA applications are characterized by excellent biocompatibility, supportive effects, and capabilities for drug loading and release [22]. Notably, IA administration of biomaterial-based therapies offers a localized treatment approach that minimizes systemic toxicity while directly targeting OA lesions [23]. The inherent properties of nanomaterials, hydrogels, and scaffolds allow for further modification and enhancement, or the synthesis of new multifunctional biomaterials by using them as carriers. Multifunctional biomaterials can achieve enhanced targeting, drug release, and modulation of the immune microenvironment components at the OA lesion site. Crucially, they achieve this without eliciting additional immune responses against the biomaterial itself. For instance, by modifying biomaterials with receptors or ligands that correspond to immune cells or cytokines present at the OA site, we can enhance targeting and improve the therapeutic efficacy of OA therapy. Additionally, combining two biomaterials (e.g., nanohydrogels) integrates their benefits while offsetting individual limitations, yielding a synergistic immunomodulatory platform [24,25].

OA causes significant damage to cartilage, which weakens the ability of MSCs in the body to repair tissues and promote cartilage regeneration. This damage, compounded by inflammatory attacks, accelerates cartilage decomposition and leads to irreversible pathological changes [26,27]. Immune-mediated biomaterials offer a promising approach to mitigating these effects by effectively inhibiting inflammation progression [28]. They achieve this by promoting the transition of macrophages from a pro-inflammatory to an anti-inflammatory phenotype and reducing the concentration of reactive oxygen species (ROS) in the OA environment. Additionally, the specialized design of these biomaterials enhances the cartilage-repairing capabilities of both native and introduced MSCs at the site of OA, thereby prolonging their therapeutic effects [29,30]. This strategic approach opens new avenues for OA therapy, shedding light on innovative therapeutic possibilities for the future.

This review provides a comprehensive analysis of the immunological mechanisms underpinning OA and explores the latest developments in biomaterial-based immunomodulation. We highlight recent progress in the design of nanomaterials, hydrogels, and scaffolds as immunoregulatory platforms for OA therapy. Furthermore, we emphasize the critical interplay between OA pathophysiology and bone immunology, advocating for a paradigm shift in OA therapeutic strategies that prioritizes immune-mediated interventions. By elucidating the intricate connections between biomaterials and immune modulation, this review serves as a foundation for future investigations aimed at advancing OA therapy.

## 2 Pathophysiology and clinical therapies of OA

### 2.1 Structural basis of the immune response in OA

Cartilage is primarily a tissue without arteries, nerves, or lymphatic vessels that absorb and buffer pressure. It is usually composed of 95 % extracellular matrix (ECM) and 3–5 % chondrocytes [31]. It has been reported that the ECM can direct and control cellular activity and play a key role in chondrocyte homeostasis [32]. Mechanical stress and inflammatory stimuli trigger an upregulation of chondrogenic polymerase and collagenase activity, accelerating ECM degradation [33]. When ECM degradation surpasses synthesis, irreversible denaturation occurs. Repairing this damage remains challenging due to the limited proliferative and synthetic capacity of mature chondrocytes. On the one hand, extracellular heparan sulfate 6-O endosulfatase (Sulf) 1 and Sulf-2 promote the release of growth factors and the development of degenerative chondrocytes [34]. On the other hand, chondrocytes proliferate and produce ECM to promote wound healing and eventually develop into large spherical hypertrophic chondrocytes. The cartilage defect in osteochondral fractures extends deep into the subchondral bone. To produce fibrous cartilage repair, mainly type I collagen, the defect is filled with bone marrow mesenchymal stem cells (BMSCs), inflammatory cells, and a range of cytokines [35]. However, fibrocartilage is prone to degenerative changes, which can lead to OA.

Beyond ECM degradation, vascularization of the subchondral bone plays a pivotal role in OA pathogenesis. Increased expression of angiogenic factors such as vascular endothelial growth factor (VEGF) and platelet-derived growth factor β in the subchondral bone promotes neovascularization [36]. Hypoxia at the inflammatory sites further amplifies VEGF production, thereby reinforcing angiogenesis. Macrophages and other inflammatory cells contribute to this process by secreting VEGF and TNF-α, which in turn upregulate matrix metalloproteinases (MMP-9 and MMP-14), facilitating vascular invasion into the ECM [37]. This bidirectional interaction between inflammation and angiogenesis perpetuates chronic inflammation and exacerbates OA progression.

### 2.2 Immune regulation in OA progression

#### 2.2.1 Immune cells

The immune cells involved in the repair of cartilage injury mainly include macrophages, osteoclasts, T cells, B cells, natural killer (NK) cells and dendritic cells (DCs) [38]. The first immune cells recruited during injury are neutrophils, which secrete proinflammatory mediators and elastases and have the ability to recruit macrophages, DCs, and NK cells. Chondrocyte apoptosis and ECM degradation can also be induced. Activated NK cells release interferon-γ (IFN-γ), and Th1 cells polarize infiltrating macrophages into M1 macrophages. Next, proinflammatory factors secreted by M1-type macrophages interfere with chondrogenic differentiation of MSCs. DCs induce Th1 and Th17 cell activation leading to cartilage degeneration, while mast cells promote ECM degradation [39]. During the repair process, macrophages are polarized into M2-type macrophages by IL-4 secreted by Th2 cells [40]. M2 macrophages can secrete anti-inflammatory factors and chondroblast cytokines, inhibit inflammation and promote cartilage repair [41]. DCs promote the chondrogenic differentiation of MSCs and induce the proliferation of regulatory T (Treg) cells by secreting IL-10. Treg cells further enhance chondrogenesis by suppressing inflammation through IL-10 and TGF-β1 secretion [42]. Moreover, NK cells play dual roles by stimulating MSC recruitment and osteoclast differentiation. Neutrophil-derived extracellular vesicles have also been shown to modulate anti-inflammatory responses, highlighting their potential role in OA resolution [21].

#### 2.2.2 Cytokines

Cytokine dysregulation is a hallmark of OA pathogenesis, creating a pro-inflammatory microenvironment that perpetuates joint degeneration. The imbalance between pro-inflammatory and anti-inflammatory cytokines initiates a vicious cycle of cartilage damage and synovial inflammation [43]. The most important inflammatory mediators in the pathogenesis of OA are IL-1β, TNF-α, and IL-6. These cytokines activate multiple signaling pathways that trigger pathological cascades. Chemokines amplify this process. Upon cytokine stimulation, they recruit inflammatory cells to joints, perpetuating inflammation and disease progression [44].

### 2.3 Clinical challenges in OA management

In the early stage of OA, drug therapy is the main treatment. Non-steroidal anti-inflammatory drugs (NSAIDs) are the most widely used drugs that reduce the production of inflammatory factors and exert analgesic effects [45]. NSAIDs relieve symptoms and reduce suffering but do not slow OA progression. However, they carry risks of gastrointestinal, neurological, hematologic, and allergic side effects [46]. Glucocorticoids, another common intervention, offer potent anti-inflammatory effects and reduce synovial effusion [47]. However, chronic use accelerates cartilage and subchondral bone degradation, limiting their clinical utility [48]. Hyaluronic acid (HA) is a component of joint fluid and cartilage matrix and is commonly used for IA injections to treat OA [49]. HA treatment restores synovial fluid viscoelasticity, enhances lubrication and shock absorption, reduces joint friction and MMP production, relieves pain, and prevents cartilage degradation. These effects deliver significant clinical efficacy [50]. HA intra-articular injections remain controversial due to surgical complexity and short-term risks like joint edema or infection [51].

In advanced OA, severe joint deformity renders medical therapy ineffective and surgical treatment is the only option, most commonly arthroscopic debridement and total joint replacement (TJR) [52]. Arthroscopic debridement is indicated for mild to moderate OA and generally provides more satisfactory results [53]. TJR remains the gold standard for end-stage OA, offering pain relief and functional restoration [54]. Although surgery can achieve effective treatment results, it is expensive and may lead to serious postoperative complications and medical risks. For example, total knee replacement doubles venous thromboembolism risk. Postoperative complications like coagulopathy, electrolyte disturbances, and pulmonary issues drive this increased mortality [55,56].

**Fig. 1:**
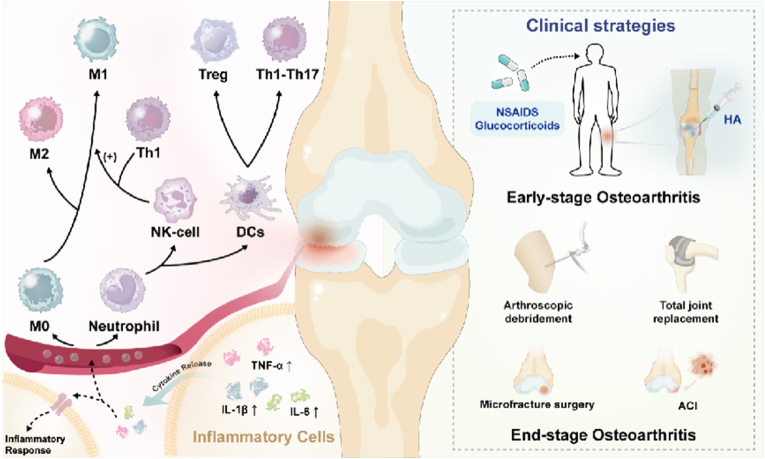
**Pathophysiology and clinical therapies of OA.** The immune cells involved in OA highlight M1 and M2 macrophages, with M1 promoting inflammation via Th1 activation and M2 exerting anti-inflammatory effects. Other immune cells, including DCs, NK cells, neutrophils, and Tregs, contribute to disease progression. Elevated levels of TNF-α, IL-1β, and IL-6 indicate an intensified inflammatory state in joint tissues. The treatment strategies of OA divide into two parts according to the clinical stages. Early-stage interventions include NSAIDs, glucocorticoids, and hyaluronic acid (HA) to reduce inflammation and improve joint function. In advanced cases, surgical options such as arthroscopic debridement, total joint replacement, microfracture surgery, and autologous chondrocyte implantation (ACI) address structural damage. The progression from pharmacological to surgical treatments reflects the disease's worsening nature and the need for stage-specific management strategies.

In addition, recruitment of bone marrow cells through microfracture to form smooth and strong repair tissue can also replace the role of cartilage. The recovery of microfracture surgery is directly related to the patient's own repair ability. The efficacy of microfracture surgery is significant for young and low-weight patients, but it is not effective for elderly and obese patients. After surgery, patients need to recover with the aid of a stent for 12 months or more. With the development of *in vitro* culture technology, autologous chondrocyte implantation (ACI) can better regenerate normal articular cartilage structure [57]. ACI allows implantation of mature and active hyaline cartilage in a single procedure while avoiding immune rejection. But ACI technology is still in its infancy, and lab-grown chondrocytes generally imply higher treatment costs [58]. Overall, due to the shortcomings and limitations of existing clinical treatment strategies, there is an urgent need to develop effective, multifunctional, targeted, and responsive OA therapies. In response to this need, various immunomodulatory biomaterial strategies have been explored. We next examine three major classes of such biomaterials – NPs, hydrogels, and scaffolds – beginning with NP-based approaches (Fig. 1).

## 3 Nanoparticles

NPs are materials with at least one dimension in the nanometer scale (1–100 nm) in three-dimensional (3D) space. They possess unique characteristics such as small size, adjustable physical and chemical properties of their surface, target specificity, and biocompatibility [59]. Due to these advantages, NPs can positively impact OA in the following ways: (1) Enhanced cellular targeting: NPs can be functionalized via covalent conjugation or physical adsorption to achieve selective targeting of immune cells, synovial cells, and chondrocytes in the OA joint cavity [60]; (2) Controlled drug release: Nanomaterials enable controlled drug release through multifunctional encapsulation. They deliver anti-inflammatory and bioactive agents directly into joints, achieving targeted sustained release while promoting tissue repair [[61], [62]]; (3) Biomimetic Strategies for Immune Modulation: NPs can be engineered to mimic native tissue microenvironments, improving their biocompatibility while actively modulating immune responses to mitigate OA-induced inflammation [63].

Nanotechnology promotes biomaterials to show great potential in improving targeting, sustained release and enhancing the efficacy of OA. However, the research of nanotechnology-boosted biomaterials in the treatment of OA is mainly in the preclinical stage and has not been widely applied in clinical practice. The safety and regulatory issues related to nanotechnology-boosted biomaterials are major challenges to be solved for successful clinical transformation. Therefore, the development of effective strategies to evaluate the safety and effectiveness of nanoparticles is crucial for their successful clinical transformation [64]. In addition, *Brown* et al. speculated that the protein crown formed by the adsorbed synovial protein may mask the functional ligands on the surface of nanoparticles [65]. Therefore, how to overcome the interference of joint synovial fluid on biomaterials is also a huge challenge for clinical transformation. Further tests in a variety of joint tissues or large animals are needed to understand and confirm the efficacy of OA [66].

In the following sections, we explore the diverse roles of NPs in OA therapy, emphasizing their applications as drug carriers, targeting systems, and bioactive therapeutic agents.

### 3.1 Nanoparticles as drug delivery carriers

**Fig. 2:**
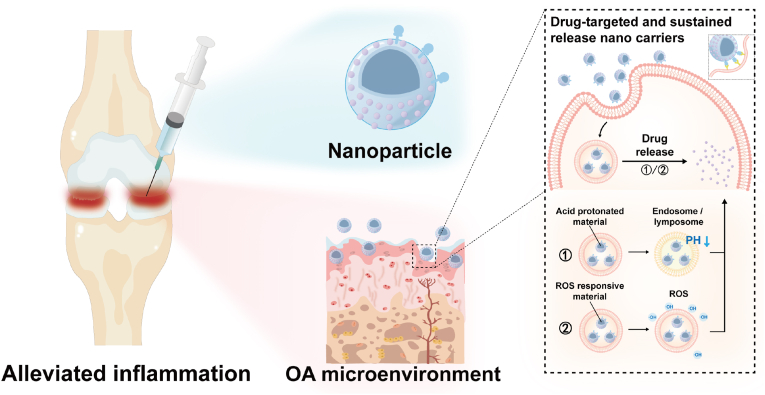
**The mechanism of common nano-drug delivery system.** The drug-loaded nanoparticles can be combined with target cells by surface modification and other means, and the drug can be accurately delivered to the target cells. To achieve sustained drug release and to regulate drug release by pH change and oxidative stress imbalance response.

Currently, the primary clinical methods for administering drugs for OA are systemic administration and IA injection [67]. While systemic administration is relatively straightforward, it faces multiple limitations. These includes gastrointestinal metabolism poor bioavailability requiring frequent dosing [68], and adverse reactions in non-target organs. Another issue is the unique avascular anatomical structure of cartilage that impedes systemic drug delivery [69]. IA injection addresses some of these issues by delivering drugs directly to the damaged site, thereby reducing systemic side effects, improving bioavailability, and better controlling drug concentration. However, IA injection faces challenges such as drug metabolism issues, poor patient compliance with joint cavity injection, and a propensity for infection, which limits its clinical application [70]. NP carriers exhibit multifunctionality in aspects like size, shape (spherical, rod, or cubic), composition, and surface charge. These features enable them to interact with the joint cavity's microenvironment while releasing drugs. As a result, they can effectively reduce systemic toxicity and achieve controlled drug release. Additionally, the use of vesicles or polymer-based drug delivery systems can protect drugs from degradation and reduce efflux from the joint cavity [71]. As illustrated in Fig. 2, we summarized the drug release mechanisms of common nano-drug delivery system and the following sections will discuss in detail the application strategies of NP carriers in the treatment of OA.

#### 3.1.1 Sustained-release nanocarriers

Nanomaterial carriers enhance joint drug retention and improve stability. This reduces therapeutic doses, decreases administration frequency, boosts efficacy, minimizes off-target toxicity, and lowers immune attack on drugs [72]. *Chang* et al. developed a novel OA therapeutic formulation, HA-liposome-diclofenac/DEX, to combat joint pain. This formulation achieves effective concentrations of DIC and DEX in a short period while allowing sustained release of DIC and DEX over the long term [73]. *Liu* et al. prepared mesoporous polydopamine nanoparticles (DAMM NPs) doped with arginine and manganese (Mn) ions to load DEX [74]. DAMM NPs sustain dexamethasone (DEX) release to suppress synovial inflammation. Additionally, their T1-T2 magnetic contrast enables MRI-traceable drug delivery. *Sangsuwan* et al. developed poly (lactic-co-glycolic acid) microspheres encapsulating flavopiridol. The strategy aims to mitigate post-traumatic OA-related inflammation by inhibiting cyclin-dependent kinase 9 (CDK9) [75]. Compared to soluble flavopiridol, flavopiridol microspheres demonstrated significant joint retention, confirming the sustained-release behavior of the microspheres. Nanocarrier sustained release systems can effectively reduce dosing frequency and decrease drug side effects.

In addition to achieving sustained release of OA drugs, NPs can encapsulate enzymes, RNA, and other active components to target the damaged site for treatment. To address the poor retention and rapid inactivation of native superoxide dismutase (SOD), *Gui* et al. utilized a highly efficient antioxidant NP system based on SOD-loaded porous polymer NPs (SOD-NPs). The synthesized SOD-NPs were capable of delivering SOD to the knee joints of mice, thereby extending the retention time in the joints [76]. Similarly, *Deng* et al. explored an anti-inflammatory treatment using mRNA encoding interleukin-1 receptor antagonist (IL-1RA) in a rat model of temporomandibular joint OA. Their team employed composite nanomicelles to encapsulate the mRNA, which provided excellent tissue penetration and minimized immunogenic responses [77].

NP encapsulation can also enhance drug delivery methods, thereby facilitating the exploration of emerging therapeutic mechanisms. *Liang* et al. prepared and characterized a melatonin nanoparticle delivery system (MT@PLGA-COLBP) [78]. MT@PLGA-COLBP uses PLGA NPs designed to reduce drug clearance efficiency and improve site-specific delivery, thereby enhancing efficacy and reducing side effects [79,80]. This design enables stable melatonin release and action. Mechanistically, melatonin inhibits innate immune system activation by suppressing the toll-like receptor 2/4 (TLR2/4)- myeloid differentiation primary response 88-NF-κB signaling pathway and scavenging ROS. These effects improve cartilage matrix metabolism and slow OA progression *in vivo*.

#### 3.1.2 Drug targeting nanocarriers

**Fig. 3:**
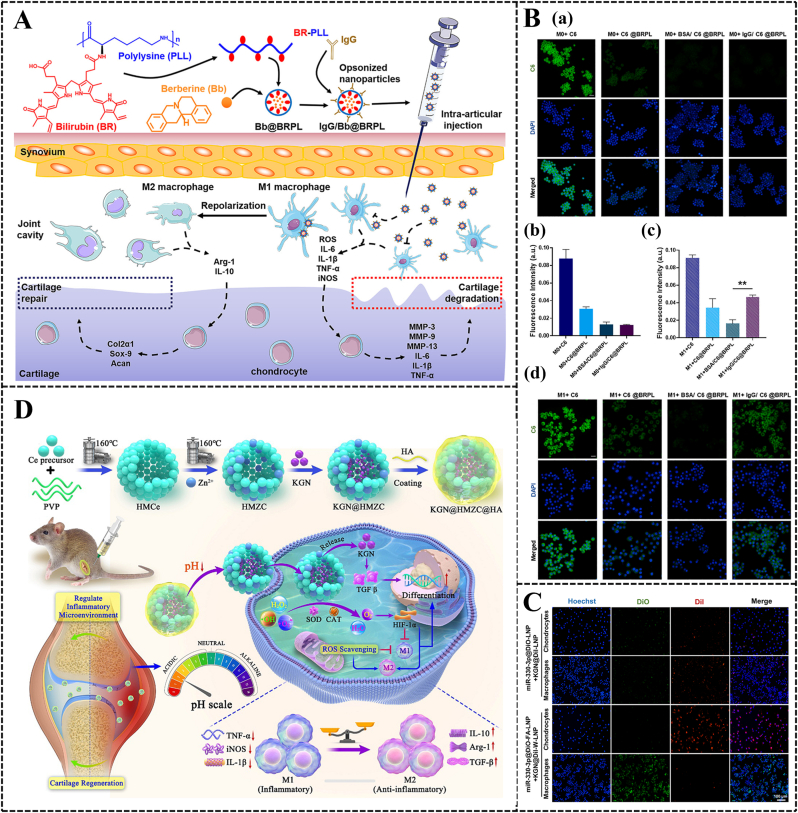
**Immunomodulation-based nanomaterials for OA therapy.** (A) Schematic diagram of the preparation of conditioning NPs IgG/Bb@BRPL and their application in OA therapy. Copyright 2022, Elsevier [86]. (B) Cellular uptake of IgG/C6@BRPL in M0 and M1 macrophages. Fluorescence images showing the uptake characteristics of free C6, C6@BRPL, BSA/C6@BRPL and IgG/C6@BRPL in (a) M0 and (c) M1. Results of (b) M0 and (d) M1 were quantified using Image J (n = 3). Copyright 2022, Elsevier [86]. (C) Chondrocytes 6 h after transfection with miR-330-3p@DiO-LNP and KGN@DiI-LNP, miR-330-3p@DiO-FA-LNP and KGN@DiI-W-LNP and representative images of macrophage uptake. Copyright 2024, Elsevier [88]. (D) Schematic of KGN@HMZC@HA nano-enzymes preparation with the specific mechanism of microenvironmental remodeling for the treatment of OA. Copyright 2024, Elsevier [89].

Nanomaterials modified with targeting components can serve as carriers to deliver drugs directly to disease sites. The receptor-ligand binding design is a classic approach in this regard. Studies have shown that the ligand WYRGRL (Coll-II α1 chain binding peptide) can specifically bind to collagen II, which is specific to the cartilage matrix [81]. Based on this characteristic, *Xiong* et al. conjugated the cartilage-targeting peptide to polyethylene glycol (PEG)-modified traditional Chinese herbal medicine, formononetin (FMN), to enhance its targeting ability to cartilage tissue [82]. *In vitro*, chondrocyte uptake experiments indicated that FMN-PEG NPs conjugated with the cartilage-targeting peptide (labeled with DID) were more readily taken up by chondrocytes compared to NPs without the targeting peptide. Furthermore, IA injection of these NPs into OA rats showed strong joint retention *in vivo*, as evidenced by fluorescence imaging. Certain physiological processes within the body can be leveraged to design drug nanocarriers for targeted regulation. The efficacy of *in vivo* treatments is often compromised due to clearance by the reticuloendothelial system (RES), also known as the macrophage system [83]. Opsonins play a crucial role in the specific recognition and uptake of exogenous microbes or NPs by macrophages in the RES. These opsonins include immunoglobulins, complements (C3, C4, C5), C-reactive proteins, laminins, fibronectins, and lectins. They bind to exogenous microbes or NPs through random Brownian motion in the bloodstream, leading to opsonization. The opsonized NPs are subsequently captured and phagocytosed by macrophages in the RES. In OA therapy, opsonized NPs can target macrophages effectively. Activated M1 macrophages have high-expression surface receptors (such as complement receptors and Fc receptors), which can capture opsonized NPs. This mechanism enhances targeting accuracy and reduces drug side effects [84,85]. Building on this mechanism, *Kou* et al. designed NPs using immunoglobulin G (IgG) as an opsonin layer to encapsulate anti-inflammatory berberine within a ROS-responsive bilirubin-grafted polylysine biomaterial core [86] (Fig. 3A). Using coumarin-6-labeled NPs, fluorescence detection showed higher uptake of opsonized NPs by M1 macrophages than M0 macrophages, demonstrating targeted M1 macrophage delivery. This targeted delivery facilitated the anti-inflammatory effects of berberine and promoted macrophage phenotype switching, thereby aiding in OA therapy (Fig. 3B). Additionally, folic acid (FA) and the overexpressed folate receptors on M1 macrophages are often used as ligand-receptor pairs for targeting macrophages. *Huang* et al. reported an M1 macrophage-targeted delivery system based on FA-modified metal-organic frameworks (MOFs), named Bai@FA-UIO-66-NH2 [87]. This system gradually released the loaded antioxidant baicalin (Bai) at a subcellular level, reducing ROS production and promoting M2 macrophage polarization, thus alleviating synovial inflammation in OA joints. To address the clinical challenges of insufficient inflammation suppression, inadequate cartilage repair, and non-targeted treatment in IA drug injections, *Yang* et al. developed novel dual-targeting lipid nanoparticles (LNPs). These LNPs encapsulate miR-330-3p (an important anti-inflammatory agent) and kartogenin (KGN, a small molecule promoting chondrogenesis). They modified the LNPs surface with FA and WYRGRL to achieve precise delivery to macrophages and chondrocytes [88]. The dual-targeting NPs, miR-330-3p@FA-LNP and KGN@WYRGRL-LNP (KGN@W-LNP), exhibited high bioavailability and selective uptake by active cells (Fig. 3C). By modulating the re-polarization of macrophages from the M1 to the M2 phenotype and maintaining chondrocyte homeostasis, KGN@W-LNP synergistically reduced synovial inflammation and cartilage degeneration.

Nanomaterials themselves can achieve targeting of diseased sites. In recent years, self-assembled dextran sulfate (DS) NPs have been used for the targeted treatment of rheumatoid arthritis. DS NPs specifically target activated macrophages via scavenger receptor class A (SR-A), enhancing anti-inflammatory effects and reducing adverse reactions [90,91]. Given the critical role of activated macrophages in OA [92], *She* et al. reported a novel dextran sulfate-triamcinolone acetonide (DS-TA) conjugate NP for the targeted treatment of OA. These DS-TA NPs harness the anti-inflammatory properties of triamcinolone acetonide by targeting macrophages [93]. The DS-TA NPs rapidly release TA under the action of esterases and target SR-A. This dual action inhibits the activity of activated macrophages and the expression of pro-inflammatory cytokines, ultimately improving OA progression by specifically inhibiting macrophage activation.

#### 3.1.3 Drug-responsive nanocarriers

Under normal physiological conditions, synovial fluid has an alkaline pH (approximately 7.7). However, due to the deposition of inflammatory metabolites in and around the joint tissues, the pH of OA synovial fluid can drop to around 6.0 [94]. CS is often chosen as a "gatekeeper" due to its pH responsiveness, which allows controlled drug release in different pH environments [95]. *Jin* et al. developed pH-responsive CSL@HMSNs-Cs (CHC) nanocarriers by assembling celastrol into hollow mesoporous silica nanoparticles (HMSNs) and coating them with chitosan (CS), creating an efficient drug delivery system [96]. Experiments showed that the drug delivery system achieved maximum release efficiency at pH 6.0. By exploiting the pH changes in the OA microenvironment, CS degrades in acidic conditions, enabling the controlled release of celastrol at the OA site, effectively addressing celastrol's poor water solubility and potential systemic toxicity [97]. Dendritic mesoporous silica nanoparticles (DMSNs) consist of a silica core enveloped by a dendritic structure, featuring a large surface area and intricate pore network. This unique structure makes DMSNs suitable for drug delivery, catalysis, and sensing, thereby enhancing drug efficacy and reducing side effects [98]. Introducing new chemical bonds during synthesis can impart responsive characteristics to DMSNs [99]. Consequently, *Jin* et al. selected pH-dependent DMSNs to encapsulate enzyme clusters [100]. They loaded the enzyme clusters into DMSNs to assemble a pH-responsive system (PEG-DMSNs-NH2@AuCu, CDP). The mildly acidic environment of OA triggers the pH-dependent release of cluster enzymes, which exert their antioxidative properties by significantly reducing ROS and stabilizing cartilage-protective proteins through the inhibition of the Hippo pathway. Bisphosphonates (BPs) are the most commonly used drugs for the treatment of many diseases that lead to abnormal bone turnover. They can reverse the subchondral bone changes of OA by inhibiting the activity of osteoclasts. *Geng* et al. prepared a pH-responsive BP-conjugated nano-apatite system by the hydrothermal method and in-situ adsorption method, in which nano-apatite is the carrier of BPs [101]. The system showed rapid and mild BP release behavior at relatively low and high pH, respectively. It can effectively inhibit the bone resorption activity of osteoclasts, inhibit the abnormal remodeling and excessive upward invasion of subchondral bone, so as to reduce OA.

In addition to pH changes, inflammatory responses in OA joints largely depend on activated macrophages, which secrete substantial amounts of ROS [102], Oxidative stress caused by ROS is a critical factor in the progression of OA [103]. Based on this insight, *Xiong* et al. synthesized a platform based on a convertible antioxidant polymer for ROS-responsive delivery of astaxanthin (ASTA) to effectively treat OA [104]. Under high ROS conditions, the main chain of the NPs, composed of poly (ethylene glycol)-poly thioester-poly (ethylene glycol), breaks down, significantly reducing ROS levels. Furthermore, ASTA is released in response to ROS levels, and the degradation products of the polymer (PEG, acetone, and thiol) are non-toxic to tissues. Flow cytometry and the Amplex Red assay results demonstrated that the synergistic effects of the NP main chain groups and the ASTA drug exhibited excellent ROS scavenging effects both intracellularly and extracellularly. Similarly, poly (ethylene glycol) diacrylate-1,2-ethanedithiol (PEGDA-EDT) copolymers can also exhibit ROS responsiveness. *Wu* et al. developed a ROS-responsive polylactic acid (PLA)/PEGDA-EDT@reduced graphene oxide (rGO)-fucoxanthin nanofibrous membrane as a delivery system for OA therapy. In this system, PLA serves as the scaffold, PEGDA-EDT functions as the ROS-responsive group, and rGO acts as the drug carrier, aiming to achieve intelligent responsiveness and subsequent long-term sustained drug release [105].

Secretory phospholipase A2 (sPLA2) is a group of heterogeneous enzymes. These enzymes specifically recognize and catalyze the hydrolysis of the SN-2 ester bond in glycerophospholipids. This catalysis releases free fatty acids like arachidonic acid (AA) and lysophospholipids. Both are well-known mediators of inflammation and tissue damage [106]. Under pathological conditions, sPLA2 can be induced by various cascade reactions and effector molecules, including inflammatory cytokines and free radicals [107]. High expression levels and activity of sPLA2 have been observed in the synovium, synovial fluid, and articular cartilage of patients with OA [108]. Based on these findings, *Wei* et al. developed NPs loaded with lipid-based sPLA2 inhibitors to suppress sPLA2 enzyme activity [72]. The lipid moiety of the inhibitor is cleaved under high concentrations of PLA2, releasing the inhibitor and thereby mitigating the inflammatory response in OA.

### 3.2 Bioactive nanoparticles in OA therapy

The inherent properties of NPs such as size, shape, charge, surface modification, stability, biocompatibility, and the ability to incorporate therapeutic agents, making them well-suited for medical applications [109]. Currently, liposomes, poly (lactic-co-glycolic acid) NPs, dendrimers, gold NPs, micelles, polymer NPs, and microneedles are extensively studied for targeting specific immune cells or immune responses [110]. Numerous biomaterials have been researched to target specific immune cells and elicit particular immune responses [111,112]. Below, we exemplify how NPs exert their unique characteristics to intervene in OA inflammatory responses.

#### 3.2.1 Anti-inflammatory/antioxidant nanoparticles

Certain NPs possess chemical compositions that inherently counteract oxidative stress and inflammatory responses. For example, *Ren* et al. designed cerium oxide nanoparticles to eliminate ROS in synovial cells, thereby alleviating inflammation, inhibiting the release of matrix-degrading enzymes, and promoting cartilage matrix synthesis [113]. Similarly, polydopamine (PDA) is a novel multipurpose biopolymer formed by the self-polymerization of dopamine, known for its excellent biocompatibility and ROS-scavenging ability. *Wang* et al. introduced an antioxidative/anti-inflammatory dual-task NP platform based on PDA NPs for treating temporomandibular joint OA [114]. Besides directly reacting with ROS as a reductant, PDA NPs may also enhance mitochondrial respiration efficiency, reduce ROS production and provide a dual antioxidative mechanism. Lignin, a natural antioxidant biopolymer, shows substantial potential in biomedical applications. *Liang* et al. grafted PLA onto lignin via ring-opening polymerization, followed by electrospinning to prepare nanofibers [115]. These PLA-lignin nanofibers serve as antioxidative tissue engineering scaffolds, protecting BMSCs from oxidative stress and promoting chondrogenic differentiation.

Nanozymes, nanomaterials with intrinsic enzyme-like activities, can efficiently catalyze enzymatic substrates under mild conditions, mimicking natural enzyme kinetics and catalytic efficiency [116]. Unlike biological enzymes, stable nanozymes can overcome issues of enzyme inactivation and rapid consumption. *Wang* et al. designed a manganese oxide (Mn_3_O_4_) nanozyme with SOD-like and CAT-like activities. The Mn_3_O_4_ nanozyme can reprogram metabolic imbalances and uncontrolled inflammatory responses induced by hydrogen peroxide (H_2_O_2_) in chondrocytes [117]. *Guo* et al. developed zinc-doped hollow mesoporous CeO_x_ (HMZC) via a simple two-step method to efficiently encapsulate KGN, subsequently wrapping HA as a "gatekeeper" on the HMZC surface, generating KGN@HMZC@HA nanozymes [89]. Due to the formation of additional oxygen vacancies during zinc doping, KGN@HMZC@HA exhibits SOD-like and CAT-like catalytic activities. These activities scavenge ROS, generate O_2_, convert M1 macrophages to M2 phenotypes, inhibit inflammation, and enable controlled KGN release in OA microenvironments (Fig. 3D).

Apart from ROS, cellular free DNA is also a risk factor in OA pathogenesis. To simultaneously clear both, *Shi* et al. prepared polyethylenimine-functionalized diselenide-bridged mesoporous silica NPs with high nucleic acid-binding and antioxidative properties [118]. This cationic material can reduce free DNA levels and oxidative stress in the joint cavity during OA progression, inhibiting M1 macrophage polarization.

#### 3.2.2 Nanoparticles with targeting capabilities

The chemical composition of nanomaterials can interact with receptors at lesion sites in various ways, achieving effective accumulation at the disease site and exerting therapeutic effects for OA. Cluster of differentiation 44 (CD44) is a type I transmembrane receptor protein. It is abundantly present in human articular chondrocytes and promotes cell proliferation by binding to HA [119]. HA-NPs have been widely studied as targeted, long-acting drug carriers that actively target pathological sites expressing HA receptors (especially CD44) [120]. *Kang* et al. used *in vitro* and *in vivo* models to show that empty HA-NPs (drug-free) have therapeutic potential for OA. HA NPs interfere with the interaction between fragmented low-molecular-weight HA and CD44, a process involved in OA pathogenesis and progression [121]. However, the short half-life of HA NPs due to the action of hyaluronidase *in vivo* limits their clinical application. Additionally, HA cannot self-assemble without the chemical conjugation of hydrophilic parts, and its large-scale production is difficult due to the complexity of the required steps [122]. To overcome these limitations, *Cho* et al. developed Levan NPs to block CD44 [123]. Levan is a polysaccharide comprising β-D-fructose polymers, where fructose rings are linked via β-glycosidic bonds. Its amphiphilic nature enables the formation of micelles through self-assembly in aqueous solutions. Levan also possesses antioxidative, antiviral, and anti-inflammatory properties [124]. *In vivo* experiments demonstrated that Levan NPs alleviate OA progression by regulating NF-κB and inhibiting CD44-induced COX2 expression. In inflammatory joint regions, activated macrophages express high levels of folate receptors, whereas their expression is low in normal cells. Utilizing this characteristic, biocompatible materials conjugated with folate have been developed to enhance targeting efficiency and therapeutic effects [125,126]. Carbon dots (CDs), a type of nanomaterial, exhibit excellent biocompatibility, small size, ease of preparation, low cost, and superior optical properties [127]. Leveraging these properties, *Jin* et al. synthesized novel folate-conjugated CDs via hydrothermal methods. The CDs target activated macrophages in inflamed joints and modulate inflammation and polarization in lipopolysaccharide (LPS)-induced macrophage models [128].

NPs can also target organelles, thereby modulating organelle functions in immune cells to block inflammatory pathways and alleviate OA inflammation. For immune cells such as macrophages, mitochondria are crucial not only for biosynthesis and energy metabolism but also for establishing and maintaining macrophage phenotype and function [129]. Mitochondrial calcium ion (m[Ca^2+^]) homeostasis is essential for normal mitochondrial function and the inflammatory phenotype of macrophages. m[Ca^2+^] overload can activate the pro-inflammatory phenotype of macrophages via excessive m[Ca^2+^] influx, increasing macrophage glycolysis, enhancing ROS production, and impairing mitochondrial buffering capacity [130]. Targeting this pathology, *Lei* et al. designed mesoporous silica nanoparticle-amidated (MSN)-ethylenebis (oxyethylenenitrilo)tetraacetic acid (EGTA)/triphenylphosphine (TPP)-polyethylene glycol (PEG) [METP] NPs to regulate m[Ca^2+^] and macrophage phenotype [131]. METP NPs can target mitochondria via TPP (a mitochondrial targeting agent) and consume excess calcium ions via EGTA (a calcium-binding group), thereby effectively and selectively inhibiting the abnormal increase in m[Ca^2+^] in macrophages of OA mice or LPS-induced cells.

#### 3.2.3 Nanoparticles combined with biomimetic strategies

Recently, cell membrane-coated NPs have emerged as a promising approach for developing functional biomimetic nanomedicines [132]. By directly replicating the complex functionalities of cell surfaces, cell membrane coatings encapsulate NPs and endow them with the innate characteristics of the source cells [133]. Various types of cell membranes are considered advantageous for coating NPs [134]. For instance, red blood cell (RBC) membranes, which have a high abundance of CD47, enable RBCs to circulate for up to four months *in vivo*, thereby providing long-circulation delivery capabilities when used to coat NPs [135]. Neutrophils, as the first responders to inflammation, can target inflamed sites by binding to Intercellular Adhesion Molecule 1 (ICAM-1) on activated endothelial cells through the CD11a ligand, thereby conferring the NPs with inflammation-targeting capabilities [136,137]. Based on this principle, *Xue* et al. developed a method to prepare neutrophil-RBC hybrid biomimetic materials by fusing neutrophil and RBC membranes and coating these on hollow copper sulfide NPs loaded with dexamethasone phosphate (D-CuS@NR NPs). D-CuS@NR NPs achieved synergistic therapy through mild heating, extended circulation, and targeted delivery [138]. Particularly, due to cell membrane coating and photothermal-responsive drug release under near-infrared irradiation, these biomimetic NPs exhibited significant cell compatibility and anti-inflammatory capabilities. Macrophage membrane-coated NPs demonstrate high targeting delivery efficiency and have shown therapeutic effects in inflammatory diseases such as rheumatoid arthritis, cancer, and sepsis [139]. *Zhou* et al. prepared biomimetic NPs coated with M2 macrophage membranes to target OA by inducing the repolarization of M1 macrophages to M2 phenotypes, thereby offering targeted treatment for OA [140].

Combining biomimetic strategies with nanoparticles enables cutting-edge osteoarthritis therapy. This approach harnesses the natural functionalities of various cell types to develop sophisticated nanomedicines capable of precise intervention in inflammatory processes. The success of such biomimetic NPs in preclinical models underscores their potential to revolutionize OA therapy and other inflammatory diseases, paving the way for more effective and personalized treatment options.

## 4 Hydrogel

Hydrogels, characterized by their 3D networks of hydrophilic polymer chains in water-rich environments, exhibit a wide range of tunable physical and chemical properties [141,142]. Their high water content facilitates the diffusion of materials, making them particularly suitable for biomedical applications [143]. Notably, hydrogels possess mechanical properties akin to articular cartilage, with a compressive modulus of 0.7–0.8 MPa, a shear modulus of 0.69 MPa, and a tensile modulus ranging from 0.3 to 10 MPa. The properties of hydrogels underscore their potential role in addressing articular cartilage-related diseases [144]. Furthermore, hydrogels mimic the ECM due to their elasticity and flexibility, providing essential structural and biochemical support to surrounding cells [145]. Their high biodegradability ensures they do not impose a metabolic burden [146]. By incorporating specific functional groups, hydrogels can achieve condition-responsive sol-gel phase transitions and exhibit injectability, making them ideal for use in restricted anatomical environments such as joint cavities [147].

Cartilage organoids (CORG) is a special organ constructed *in vitro* using tissue engineering technology. It has a similar structure and function with cartilage tissue, but its application prospects are far beyond cartilage repair. In recent years, using hydrogel to culture stem cells and induce them to differentiate into chondrocytes has become a promising method to construct CORGs *in vitro*. Hydrogel not only simulates the ECM in natural environment, but also provides support structure and bioactive molecules for cell growth. Hydrogel-based CORG is formed by co-culturing cells with hydrogel materials. CORG can be used to create various disease models related to cartilage, assist people to understand the mechanism in cartilage genetic engineering, and can also be used in drug development, toxicity testing, and cartilage repair [148]. For example, silk fibroin (SF) is an excellent hydrogel raw material. SF hydrogel not only has ECM-like structure, but also has unique mechanical properties and significant biocompatibility. SF hydrogels based on multiple crosslinking and modification strategies have many advantages: satisfactory biological characteristics, suitable internal structure, acceptable mechanical properties, excellent cell loading capacity, and significant delivery capacity of bioactive substances. Therefore, it is considered to be an ideal biomaterial for the construction of cartilage-like organs [149].

Due to their high-water content, ECM-like structure, and adjustable mechanical properties, hydrogels have been extensively investigated as immunomodulatory biomaterials for OA therapy. The immunomodulatory potential primarily manifests in two ways: Inherent immunomodulatory properties, where hydrogels contain bioactive components that modulate immune responses; Serving as delivery vehicles, where hydrogels encapsulate immunosuppressants, anti-inflammatory antibodies, or regeneration-promoting bioactive molecules, fostering a favorable immune microenvironment. Additionally, smart-responsive hydrogels have garnered interest in OA immunotherapy, as they can be tailored to respond to specific pathological features within the OA joint microenvironment.

### 4.1 Hydrogels with special physicochemical and biological properties

**Fig. 4:**
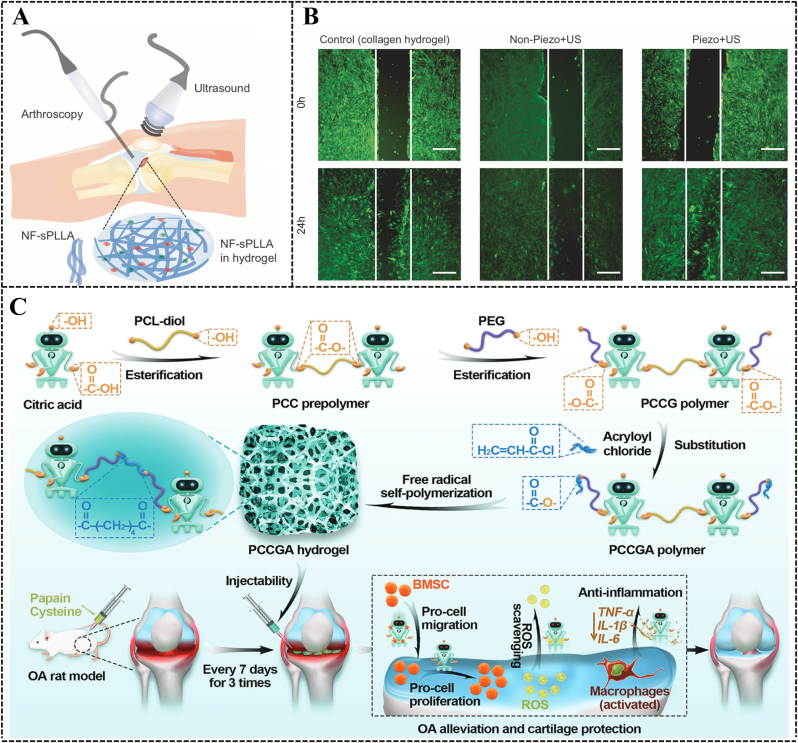
**Immunomodulation-based hydrogel for OA therapy.** (A) Schematic diagram of piezoelectric hydrogel used for OA patients. Copyright 2023, NATURE PORTFOLIO [154]. (B) Stem cell migration study assessed by scratch test, which was accomplished by filling the wound bed with Piezo, Non-Piezo, and collagen hydrogels. Copyright 2023, NATURE PORTFOLIO [154]. (C) Preparation of multifunctional PCCGA hydrogel for OA relief and cartilage protection. Copyright 2024, WILEY [159].

The unique physicochemical properties of hydrogel fractions hold significant research potential in the treatment of OA. Electrical stimulation (ES) has demonstrated notable efficacy in promoting tissue regeneration, particularly in bone and cartilage [150]. Within cartilage, electrical currents or charges are naturally produced during joint movement or deformation [151]. Consequently, external ES may aid cartilage repair by restoring the disrupted electrical microenvironment in damaged cartilage. Biodegradable piezoelectric materials, which can self-generate electricity under mechanical stresses such as joint loading or ultrasonic acoustic pressure, offer a high degree of safety. These biodegradable piezoelectric materials include synthetic polymers like poly L-lactic acid (PLLA) and amino acids such as glycine and diphenylalanine [152,153]. *Vinikoor* et al. introduced an injectable, biodegradable piezoelectric hydrogel composed of short electrospun poly-L-lactic acid nanofibers embedded in a collagen matrix. This hydrogel can be injected into joints and self-generate localized electrical signals activated by ultrasound, promoting cell migration and inducing endogenous growth factors like TGF-β1 to facilitate cartilage healing [154] (Fig. 4A–B). In addition, *Wang* et al. developed a sericin protein (silk) hydrogel using diglycidyl ether (BDDE) as a cross-linking agent [155]. The silk/BDDE hydrogel exhibited high elasticity (compression modulus of 166 ± 15.0 kPa), fatigue resistance, and stable mechanical strength in aqueous solutions. They further prepared silk/BDDE hydrogel spheres using oil/water (o/w) emulsification, which were biocompatible and suitable as a biolubricant for OA therapy. The silk/BDDE hydrogel spheres reduced cartilage damage and resisted the digestive environment of the joint cavity for extended periods. Natural polymers, such as CS, may offer advantages due to their structural, compositional, and mechanical similarities to ECM components. For instance, lactose-modified chitosan (CTL) with boronic acid cross-linking has been explored [156]. *Scognamiglio* et al. aimed to develop and characterize a CTL-boronic acid cross-linked hydrogel as a mucoadhesive for OA therapy [157]. The dynamic cross-linked polymer network of CTL and boric acid demonstrated strain-hardening behavior under increasing mechanical stress, akin to biopolymers like collagen and actin, which are integral to the mechanical transduction process [158]. This behavior can stimulate specific biological responses that ameliorate OA pathology. The CTL hydrogels' ability to act as ROS scavengers and their enhanced resistance to degradation underscore the advantages of employing such systems at the onset of OA.

A significant factor in OA's chronic inflammation is the presence of high levels of ROS, which induce oxidative stress and damage lipids, proteins, and DNA, ultimately resulting in cell damage and death. *Lei* et al. developed a bi-dynamic covalently crosslinked hydrogel, termed oHA-PBA-PVA gel (HPP), by synthesizing 3-aminophenyl boronic acid-modified HA and crosslinking it with polyvinyl alcohol (PVA) [160]. HPP forms a dynamic phenylboronate bond between phenylboronic acid from oxidized hyaluronic acid-phenylboronic acid (oHA-PBA) and the hydroxyl group from PVA, exhibiting ROS responsiveness and excellent ROS scavenging ability [161]. Similarly, *Wang* et al. reported a one-component multifunctional polycitrate-based (PCCGA) hydrogel, prepared through the self-polymerization of PCCGA polymers [159]. PCCGA possesses multifunctional properties, including injectability, adhesion, controllable pore size and elasticity, self-healing ability, and photoluminescence. Additionally, the hydrogel scavenges intracellular ROS for antioxidant effects while downregulating pro-inflammatory cytokines and promoting stem cell proliferation/migration to exert anti-inflammatory action (Fig. 4C). Epigallocatechin-3-o-gallate (EGCG), a catechin found in green tea, inherently modulates inflammation and scavenges free radicals. *Jin* et al. combined EGCG with tyramine-coupled HA and gelatin to create a composite hydrogel that protects chondrocytes from pro-inflammatory factors and promotes cartilage regeneration [162].

Macrophages, the most abundant immune cells in the synovium, play a crucial role in controlling inflammatory joints. Reprogramming pro-inflammatory macrophages into anti-inflammatory ones is essential for effective treatment [163,164]. *Huang* et al. developed immune cell-mobilizing hydrogel microspheres using HA-methacrylate and chondroitin methacrylate sulfate. The microspheres feature streptavidin-modified surfaces that recruit and capture deep synovial macrophages. This process reprograms the macrophages into anti-inflammatory phenotypes [165]. Furthermore, *Jia* et al. developed FPSOH hydrogels through the self-crosslinking of poly (salicylic acid)-F127-poly (salicylic acid) (FPS) and hyaluronic acid-3-hydroxyanthranilic acid (OH) [166]. FPS is a temperature-sensitive hydrogel, while OH is synthesized via a Schiff base reaction between aldehyde-HA and 3-HAA [167,168]. 3-HAA, a metabolite of the essential amino acid tryptophan, attenuates inflammation by modulating the conversion of macrophages from the M1 to M2 phenotype [169]. It also inhibits the expression and release of classical inflammatory signaling pathways and inflammatory factors, such as NF-κB, thereby exerting a favorable anti-inflammatory effect [170,171].

### 4.2 Multifunctional hydrogel delivery systems

Hydrogels, with their porous structure, are particularly well-suited for loading multiple substances and releasing them gradually at targeted sites [172]. Due to their non-toxic, biodegradable, and biocompatible properties under optimal conditions, hydrogels are predominantly utilized as space-filling scaffolds. These scaffolds are instrumental in transporting cells and bioactive agents, particularly in the treatment of OA [173]. Table 1 provides a summary of representative hydrogel-based delivery systems categorized by their functional classification, representative materials, delivery modality, target cells and pathways, immunomodulatory mechanisms, and application advantages.

**Table 1:** Representative hydrogel-based delivery systems.

Functional Classification	Representative Materials	Delivery Modality	Target Cells/Pathways	Immunomodulatory Mechanism	Application advantages	Ref
**Targeted delivery**	CPHs	Functionalized Nanogel	Macrophages (M1→M2), ROS, H_2_O_2_	FA/HA-functionalized nanogels bind to FR-β and CD44 receptors on activated macrophages, triggering CO release. CO modulates oxidative stress by consuming excessive H_2_O_2_, reduces ROS levels, and suppresses inflammatory cytokine secretion (IL-6, TNF-α, IL-1β)	Effectively reduces oxidative stress, inhibits inflammatory response, protects chondrocytes, and prevents cartilage degradation	[174]
	CAP/FGF18-hyEXO@HMs	Gene-Edited Exo Hydrogel Hybrid	FGF18 gene, PI3K/AKT signaling, CD44/FR-β receptors	CRISPR/Cas9-mediated FGF18 upregulation in chondrocytes enhances ECM synthesis (COL2, SOX9), suppresses pro-inflammatory cytokines (IL-1β, TNF-α), and promotes anti-inflammatory IL-10 via PI3K/AKT pathway modulation	Combines gene editing with lubrication, enhances cartilage regeneration, reduces inflammation/ECM degradation, and prolongs therapeutic effect	[175]
	GGA@Lipo@PTH (1–34) (GLP) hydrogel	Liposome-Encapsulated Hydrogel	ATDC5 cell, PI3K/AKT signaling pathway, ROS	GGA scavenges ROS, reduces inflammatory cytokines (IL-6, TNF-α), the GLP hydrogel continuously releases PTH (1-34), promotes ATDC5 cells proliferation and protects the IL-1β-induced ATDC5 cells from further progression potentially through PI3K/AKT signaling pathway	Sustained PTH 1–34 release (over 20 days), promotes cartilage repair, inhibits ECM degradation, biocompatible/non-toxic, preserves joint mobility, and reduces intra-articular injection frequency	[176]
**Controlled Release and Increased Retention Time**	O3 NPs@MHPCH	Ozone-Nanoparticle Embedded Hydrogel	Macrophages, ROS, VEGF signaling	Suppresses M1 macrophage polarization, reduces ROS and pro-inflammatory cytokines (IL-1β, IL-6, TNF-α, iNOS), inhibits VEGF secretion, and promotes anti-inflammatory responses via D-mannose synergy	Enhances O3 stability, local anti-inflammatory and cartilage protective effects, sustained drug/gas release, and promotes cartilage regeneration	[177]
	AM2M	Biomimetic Macrophage-Mimicking Hydrogel	Inflamed chondrocytes, macrophages (M1→M2); CD44, TLR4, ICAM1 receptors, MMPs, inflammatory cytokines (IL-1β, TNF-α, IL-6)	ChS release down-regulates NF-κB nuclear translocation, inhibits inflammation via CD44/TLR4/ICAM1 interaction, promotes TGF-β1 secretion for cartilage repair. Macrophage membrane shields ChS-TLR2 interaction, reducing ROS and pro-inflammatory cytokines (IL-1β, TNF-α, IL-6)	Targetes delivery to inflamed joints, on-demand drug release (burst during acute flares, sustained for repair), prolonged retention, reduces immunostimulation, decreased chondrocyte apoptosis, and inhibition of inflammatory cytokine rebound	[2]
	GelMA@Lipo@KGN	Liposome-Hydrogel Composite	BMSCs, CBFβ-RUNX1 pathway, Aggrecan/Col2a1/SOX-9 expression	KGN activates CBFβ-RUNX1 pathway in BMSCs, promoting chondrocyte differentiation and extracellular matrix production (Aggrecan, Col2a1). Sustained KGN release reduces inflammatory cytokine levels (IL-6, TNF-α) associated with OA progression	Prolongs KGN release over three weeks, enhances joint retention (>5 weeks), reduces osteophyte formation, and prevents cartilage/subchondral bone degeneration	[178]
**Enhanced Drug/Exosome Stability**	W-Exo-L@GelMA	Exo Loaded Hydrogel	Catabolic pathways (MMP3, MMP13, COX2, iNOS), anabolic pathways (COL2, SOX9, aggrecan), and inflammation/immune pathways (TNF, IL-17, NF-κB signaling)	W-Exo-L@GelMA delivers LRRK2-IN-1 via cartilage-targeting WYRGRL peptide-modified exosomeExo. LRRK2 inhibition suppresses catabolic enzymes (MMP3/13), inflammatory mediators (COX2, iNOS), and IL-1β-induced cytokine/chemokine signaling (e.g., Cxcl1, Ccl5), while promoting anabolic factors (COL2, aggrecan)	Enhances cartilage targeting and joint retention ability, reduces systemic clearance rate, has anti catabolic and pro synthetic metabolic effects	[179]
	MGS@DMA-SBMA	Microfluidic Gelatin Methacrylate Hydrogel Microsphere	Chondrocytes; TNF-α-induced inflammation, MMP13, ADAMTS5, TAC1	Hydration lubrication via zwitterionic pSBMA brushes reduces friction; sustained DS release inhibits TNF-α-induced degradation, downregulates MMP13/ADAMTS5 (cartilage catabolism), and suppresses TAC1 (pain signaling)	Enhances joint lubrication, sustained anti-inflammatory drug release, reduces microsphere degradation, excellent biocompatibility, dual therapeutic effects (mechanical protection + inflammation suppression)	[180]
**Joint Lubrication**	GelMA@DMA-MPC	Hydrophilic Polymer-Grafted Hydrogel Microsphere	Chondrocytes; IL-1β-induced inflammation, MMP13, ADAMTS5	Hydration lubrication via zwitterionic MPC brushes reduces friction; controlled DS release inhibits IL-1β-induced inflammation, downregulates MMP13/ADAMTS5, and upregulates collagen II/aggrecan (cartilage anabolism)	Improves injectability and joint dispersion, prolongs drug retention, reduces cartilage wear, synergistic lubrication-drug delivery, minimal invasiveness	[181]
**Immune Microenvironment Modulation**	BMSCs and PDGF-BB loaded GMs	MSC-Loaded Hydrogel Microspheres	Macrophages (M1→M2), IRAK4/TRAF6/NF-κB pathway, MFCs	STS drives M2 macrophage polarization by suppressing pro-inflammatory cytokines (IL-1β, TNF-α) and oxidative stress, while inhibiting IRAK4/TRAF6/NF-κB signaling to protect MFCs from inflammation-induced apoptosis/ECM degradation	Combines mechanical support and bioactive microenvironment for cell proliferation; enhances anti-inflammatory/antioxidant effects, promotes meniscal regeneration, and prevents osteoarthritis progression.	[182]

**Abbreviation List**	**1. CPHs**: A multifunctional anti-inflammatory drug based on a peptide dendrimer nanogel was constructed by physically encapsulating CO release molecules-401 and wrapping its surface with FA-modified HA **2. FA**: Folic Acid **3. HA**: Hyaluronic Acid **4. FR-β**: Folate Receptor-β **5. IL**: Interleukin **6. TNF-α**: Tumor Necrosis Factor-alpha **7. CAP**: Chondrocyte-affinity peptide **8. FGF18**: Fibroblast Growth Factor 18 **9. HMs**: Hydrogel Microspheres **10. CRISPR**: Clustered Regularly Interspaced Short Palindromic Repeats **11. Cas9**: CRISPR-associated protein 9 **12. ECM**: Extracellular Matrix **13. COL2**: Collagen Type II **14. SOX9**: SRY-Box Transcription Factor 9 **15. PI3K**: Phosphoinositide 3-Kinase **16. AKT**: Protein Kinase B **17. CD44**: Cluster of Differentiation 44 **18. GGA**: Gallic acid-grafted gelatin **19. Lipo**: Liposome **20. PTH (1–34)**: Parathyroid Hormone fragment (1–34) **21. GLP**: GGA@Lipo@PTH (1–34) hydrogel **22. O_3_**: Ozone **23. NPs**: Nanoparticles **24. MHPCH**: Mannose-conjugated HydroxyPropyl Chitin **25. VEGF**: Vascular Endothelial Growth Factor **26. iNOS**: Inducible Nitric Oxide Synthase **AM2M**: Artificial M2 Macrophage **TLR4**: Toll-Like Receptor 4 **27. ICAM1**: Intercellular Adhesion Molecule 1	**MMPs**: Matrix Metalloproteinases **28. ChS**: Chondroitin Sulfate **29. NF-κB**: Nuclear Factor Kappa B **30. TGF-β1**:Transforming Growth Factor-beta 1 **31. GelMA**: Gelatin Methacryloyl **32. KGN**: Kartogenin **33. CBFβ**: Core-Binding Factor Beta **34. RUNX1**: Runt-Related Transcription Factor 1 **35. BMSCs**: Bone Marrow Mesenchymal Stem Cells **36. OA**: Osteoarthritis **37. W-Exo-L**: WYRGRL-modified Exosomes loaded with LRRK2-IN-1 **38. LRRK2**: Leucine-Rich Repeat Kinase 2 **39. COX2**: Cyclooxygenase-2 **40. CXCL1**: C-X-C Motif Chemokine Ligand 1 **41. CCL5**: C-C Motif Chemokine Ligand 5 **42. MGS**: Microfluidic Gelatin Methacrylate Sphere **43. DMA**: Dimethylacrylamide **44. SBMA**: Sulfobetaine Methacrylate **DS:** Diclofenac Sodium **45. ADAMTS5**: A Disintegrin And Metalloproteinase with Thrombospondin Motifs 5 **46. TAC1**: Tachykinin Precursor 1 **47. MPC**: Methacryloyloxyethyl Phosphorylcholine **48. PDGF-BB**: Platelet-Derived Growth Factor-BB **49. GMs**: Hydrogel Microspheres **50. IRAK4**: Interleukin-1 Receptor-Associated Kinase 4 **51. TRAF6**: TNF Receptor-Associated Factor 6 **52. MFCs**: Meniscus Fibrochondrocytes **STS**: Sodium Tanshinone IIA Sulfonate

#### 4.2.1 Targeted delivery

The inflammatory response in OA joints is primarily driven by activated macrophages, which secrete substantial amounts of ROS intracellularly, with H_2_O_2_ being the most stable and abundant form [102]. Consequently, targeting the proliferation of activated macrophages and eliminating the excessive H_2_O_2_ they produce can significantly diminish ROS levels and mitigate inflammatory responses at affected sites. These macrophages typically overexpress folate receptor β (FR-β) and hyaluronan receptor (CD44) on their cell membranes. The corresponding ligands, folate (FA) and hyaluronan (HA), can be specifically directed to macrophages by binding them to the surface of nanogels [183]. *Yang* et al. developed multifunctional anti-inflammatory drugs (CPHs) utilizing CO gas therapy. The CPHs incorporate CO-releasing molecule-401 (COMR-401) as the donor, peptide dendrimer nanogels (PDNs) as carriers, and FA-modified HA as the targeting ligand [174]. These CPHs effectively target macrophage activation. COMR-401 rapidly releasing CO intracellularly, consuming large quantities of H_2_O_2_ and inhibiting the secretion of inflammatory factors. The CPHs provides an efficient treatment for OA. Additionally, fibroblast growth factor 18 (FGF18) plays a crucial role in cell proliferation and differentiation during cartilage formation and is a key gene implicated in the development of arthrodysplasia and OA [184]. *Chen* et al. proposed a CRISPR/Cas9-based strategy to activate the FGF18 gene in OA chondrocytes at the genomic level *in vivo*. This approach employs a chondrocyte affinity peptide-conjugated hybrid exosome loaded with FGF18-targeted gene editing tools (CAP/FGF18-hyexo) [175]. The injectable CAP/FGF18-hyEXO@HMs, combined with *in vivo* FGF18 gene editing, synergistically promote cartilage regeneration, reduce inflammation, and prevent ECM degradation.

#### 4.2.2 Controlled release

OA is primarily driven by inflammation mediated through cytokines and chemokines, which serve as the main pathophysiologic mechanism. The subsequent processes of cartilage destruction and osteoarthritic changes are secondary to this inflammation. A study by *Kim* et al. introduced a novel self-assembling peptide (SAP)-substance P (SP) hydrogel, which demonstrated significant anti-inflammatory effects, apoptosis inhibition, recruitment of intrinsic MSCs, and cartilage regeneration [185]. SP, an intrinsic substance involved in the immune response, plays a role in various biological processes, including injury perception and inflammation [186]. The SAP hydrogel forms an ECM within tissues, enhancing stem cell function by binding to target receptors, thereby increasing the residence time of SP in the joint cavity [187]. This interaction underscores the role of the ECM in modulating immune responses. Epiphycan (EPYC), a crucial ECM component, regulates the IL-17 signaling pathway in chondrocytes by modulating the interaction between IL-17A and its receptor IL-17RA [188]. This regulatory mechanism highlights the intricate interplay between the ECM and immune signaling in OA pathogenesis. Polycyclic aromatic hydrocarbons (PAH) have been shown to significantly reduce chondrocyte hypertrophy and support cartilage tissue recovery by targeting EPYC. To enhance the targeting of EPYC and prolong the *in vivo* release of PAH, *Huang* et al. developed PAH@DECM hydrogels using cartilage decellularized matrix (DECM) hydrogels as bioactive scaffolds. As DECM degrades, the released PAH is gradually utilized by chondrocytes, exerting anti-inflammatory effects and promoting chondrocyte migration and proliferation [189]. Additionally, teriparatide (PTH (1-34)) inhibits the terminal differentiation of articular chondrocytes, thereby slowing OA progression [190]. *Li* et al. incorporated a lipid-anchored PTH (1-34) into a gallic acid-grafted gelatin-injected hydrogel (GLP) GLP is a fully biodegradable and biocompatible natural polysaccharide hydrogel system designed to control PTH (1-34) release [176]. Gallic acid-grafted gelatin (GGA), an important gelatin derivative, not only retains the beneficial properties of gelatin but also possesses potential ROS scavenging and anti-inflammatory properties [191]. The IA injection of GLP, with sustained release of PTH (1-34), protects against cartilage degradation, slows OA progression, and promotes glycosaminoglycan synthesis.

Therapeutic gases have emerged as promising agents in disease treatment due to their ability to efficiently traverse cellular membranes and reach subcellular organelles, coupled with a favorable safety profile [192]. Among these, hydrogen (H_2_) has demonstrated therapeutic potential, exhibiting beneficial effects such as anti-oxidative stress, anti-inflammation, and anti-apoptosis [193]. H_2_ selectively neutralizes the most cytotoxic hydroxyl radicals among ROS, without disrupting other physiologically beneficial ROS, making it a promising candidate for OA therapy [194]. To facilitate targeted and sustained H_2_ release, *Zhang* et al. developed an injectable hydrogel platform loaded with calcium boride nanosheets (CBN@GelDA hydrogel), serving as a highly efficient and sustainable H_2_ precursor for OA therapy [195]. The CBN component enables H_2_ release under physiological conditions, while the dopamine-grafted gelatin (GelDA) substrate provides a stable microenvironment, ensuring sustained H_2_ release for over a week. Additionally, ozone (O_3_) has shown positive effects in OA therapy due to its antioxidant, anti-inflammatory, and immunomodulatory properties [196]. To achieve prolonged and stable O_3_ release *in vivo*, *Wu* et al. developed an injectable thermoresponsive hydrogel containing ozone-rich nanocomposites and D-mannose (O_3_ NPs@MHPCH). In this formulation, O_3_ is encapsulated within NPs composed of perfluorotributylamine and fluorinated HA, enhancing its stability [177]. The O_3_ NPs@MHPCH formulation improves the efficacy of O_3_ treatment, significantly alleviating OA symptoms by reducing synovial inflammation, cartilage destruction, and subchondral bone remodeling.

Nanogels (NGs) are increasingly recognized as the next generation of drug delivery systems due to their stability, high drug loading capacity, adjustable particle size, stimulus responsiveness, and ability to sustain drug release through an in-situ gel mechanism [25,197]. *Li* et al. developed glycol chitosan/fucoidan nanogels loaded with the anti-inflammatory peptide KAFAK (GC/Fu@KAFAK NGs) using electrostatic interactions and genipin cross-linking [198]. These GC/Fu@KAFAK NGs effectively inhibited the expression of inflammatory factors such as IL-6 and TNF-α, while enhancing the expression of cartilage markers like type II collagen, aggrecan, and SRY-box transcription factor 9. Macrophages critically regulate osteoarthritis progression. Specifically, M2 macrophages exert anti-inflammatory effects and promote cartilage repair [[199], [200]]. However, M2 macrophages also express low levels of certain pro-inflammatory cytokines and can induce cartilage damage through the expression of MMPs [201]. Therefore, modulation of the beneficial secretion of M2 macrophages is needed to improve OA therapy. To address this challenge, *Ma* et al. proposed the creation of an artificial M2 macrophage (AM2M), which consists of macrophage membranes as the "shell" and an inflammation-responsive nanogel as the "yolk". This NG, composed of gelatin and chondroitin sulfate (ChS), leverages the significant anti-inflammatory effects of ChS, which also has the potential to inhibit apoptosis of damaged chondrocytes and repair damaged cartilage [2]. The AM2M system is designed to mimic the beneficial secretion of M2 macrophages, achieving dual control of ChS release in response to OA inflammatory activity. During acute inflammation, bursts of ChS release can downregulate hyperinflammation through MMP-mediated degradation. Conversely, a slow and sustained release of ChS can facilitate cartilage repair and prevent the re-induction of inflammatory mediators as inflammation subsides.

#### 4.2.3 Improved stability of "goods"

Hydrogels, due to their stable structure and unique physicochemical properties, serve as effective delivery systems that enhance the stability and retention time of "cargo" such as liposomes and exosomes. This, in turn, improves the functionality of the delivery system and enhances therapeutic outcomes.

Liposomes, which are vesicles composed of phospholipid bilayers, can encapsulate hydrophilic drugs within their core or hydrophobic drugs within their lipid bilayer shell [202]. They are known for their biocompatibility, controlled drug release, and passive targeting capabilities [203]. However, liposomes often suffer from poor chemical and physical stability, leading to potential drug leakage during storage or transport. Additionally, conventional liposomes are prone to degradation and clearance by the reticuloendothelial system *in vivo*, resulting in reduced residence time [204]. Therefore, enhancing the stability and extending the *in vivo* residence time of liposomes is crucial.

Hydrogels, owing to their structural stability and biocompatibility, are particularly well suited for localized drug delivery. When combined with liposomes to form composite hydrogels, the advantages of both biomaterials are maximized [205,206]. These composite hydrogels improve the mechanical properties and stability of liposome formulations, facilitating localized and sustained drug release, which enhances the convenience and efficiency of drug delivery at the target site [207]. For instance, *Yang* et al. developed GelMA@Lipo hybrid microgels by combining liposomes with photo-crosslinkable gelatin methacryloyl (GelMA) matrices using an innovative microfluidic technique under UV light [178]. In these microgels, liposomes are securely immobilized through physical network barriers and non-covalent interactions. Encapsulated within GelMA@Lipo microgels, KGN, a hydrophobic small molecule that promotes chondrogenic differentiation of bone marrow MSCs via the CBFβ-RUNX1 pathway, demonstrated significant slow-release kinetics. Similarly, *Miao* et al. employed microfluidics to design bilayer microspheres responsive to the OA microenvironment (ChsMA + CLX@Lipo@GelMA) [208]. Initially, celecoxib (CLX) was encapsulated into liposomes using a thin-film dispersion method. Subsequently, chondroitin methacryloyl sulfate (ChsMA) microspheres were prepared via microfluidics, freeze-dried, and immersed in a CLX@Lipo@GelMA solution. The outer GelMA layer responds to MMPs and degrades rapidly in the microenvironment, releasing CLX-loaded liposomes that provide anti-inflammatory effects while lubricating. The inner core structure, containing ChsMA microspheres, is then exposed and begins to degrade, promoting cartilage repair. Furthermore, *Chen* et al. integrated cartilage affinity peptide (WYRGRL)-modified liposomes, loaded with mitophagy activator (urolithin A), into HA methacrylate hydrogel microspheres using microfluidics, resulting in HM@WY-Lip/UA [209]. This system effectively targets chondrocytes, selectively removes dysfunctional subcellular mitochondria, restores mitochondrial function, scavenges ROS, and maintains chondrocyte homeostasis by enhancing mitochondrial autophagy.

Exosomes (Exo), vesicles secreted by cells, are considered excellent vehicles for drug delivery due to their ability to penetrate natural barriers, maintain high circulatory stability, and achieve specific targeting [210]. Notably, Exo derived from MSCs or other cell types possess intrinsic properties that can reduce local inflammation, promote anabolism, and inhibit catabolism, thereby aiding in the restoration of OA cartilage [211]. However, the therapeutic efficacy and application of Exo are limited by their rapid clearance from the joint due to cellular and enzymatic removal. To address this challenge, natural hydrogels have been shown to enhance the retention of Exo, preventing their swift clearance or leakage from the joints [212,213]. In a study by *Wan* et al., an engineered Exo loaded with LRRK2-IN-1 (W-Exo@GelMA) was investigated [179]. The researchers modified the surface of the Exo with a cartilage affinity peptide to improve its targeting ability. They then encapsulated the modified Exo in a photocrosslinking spherical GelMA hydrogel. This encapsulation prevents rapid clearance and degradation. This engineered Exo, W-Exo-L@GelMA, significantly inhibited the expression of OA-associated inflammatory and immune genes and counteracted the transcriptome response induced by IL-1β. LRRK2-IN-1 is a small molecule inhibitor of leucine-rich repeat kinase 2 (LRRK2). It regulates mitochondrial homeostasis, microtubule dynamics, inflammation, and autophagy. This multi-target action positions LRRK2-IN-1 as a promising disease-modifying therapy for OA [214].

Although most studies are still at the molecular and preclinical levels, the accumulation of evidence from animal models has promoted the transition from Exo-based therapy to clinical application [215]. *Gupta* et al. have conducted several clinical trials to explore the therapeutic potential of umbilical cord (UC)-derived Wharton's jelly (WJ) for knee OA. In a prospective pilot study (NCT04719793), researchers aim to assess the 1-year outcomes in 12 knee OA patients to establish preliminary feasibility [216]. In another pivotal multicenter RCT (NCT04711304), 168 Kellgren-Lawrence grade II/III knee OA patients were randomized across 53 U S. sites to compare UC-WJ against hyaluronic acid and saline controls. The latter trial employs comprehensive 1-year efficacy/safety evaluations, encompassing patient-reported outcomes, physical examinations, and imaging analyses. Should UC-WJ demonstrate superior efficacy and safety compared to both comparators, it could potentially emerge as a breakthrough regenerative therapy [215].

#### 4.2.4 Joint lubrication

OA is a condition characterized by joint lubrication dysfunction, which correlates with the decline in joint lubrication performance, leading to progressive, irreversible damage to articular cartilage and a persistent inflammatory response [217]. Consequently, strategies that enhance cartilage lubricating properties in conjunction with anti-inflammatory therapies are deemed effective for managing OA.

Hydrogel microspheres, unlike conventional hydrogels, offer significant advantages in injectability due to their spherical shape and relatively uniform size [218]. Their fluidity and flexibility enable them to disperse effectively in solutions, making them suitable as drug carriers within joints [219]. Hydrogel microspheres crafted through microfluidic techniques present a promising option for high-performance bio-lubricants. For instance, *Yang* et al. developed a poly (dopamine methacrylamide-to-sulfobetaine methacrylate)-grafted microfluidic gelatin methacrylate sphere (MGS@DMA-SBMA), leveraging the ultra-lubricity of ball bearings and the adhesive properties of mussels [180]. These monodisperse, uniformly sized microspheres were initially prepared using microfluidic techniques, followed by spontaneous modification with a DMA-SBMA copolymer through a bionic grafting method. This process resulted in a strong hydration layer around the charged head groups (-N^+^ (CH_3_)_2_ - and -SO^3−^) of the grafted poly sulfobetaine methacrylate (pSBMA), enhancing the microspheres' lubricity. Additionally, the porous structure of the microspheres facilitated efficient drug-loading and drug-releasing capabilities. When loaded with diclofenac sodium (DS), these super-lubricated microspheres, exhibiting good biocompatibility, were able to inhibit tumor necrosis factor-α (TNF-α) induced chondrocyte degradation. Inspired by the super-lubricating properties of cartilage and the catecholamine chemistry of mussels, *Han* et al. also explored the enhancement of hydrogel microspheres' lubricating properties [181]. They incorporated a self-adhesive polymer (free radical copolymerized into DMA-MPC) onto the surface of photo-crosslinked methacrylate gelatin hydrogel microspheres (GelMA prepared by microfluidics). The microspheres were then coated with diclofenac sodium, an anti-inflammatory drug, to create lubricated GelMA@DMA-MPC microspheres.

#### 4.2.5 Constructing a suitable immunization microenvironment

In the inflammatory microenvironment characteristic of OA, MSCs often undergo aberrant differentiation, which impairs their ability to fully utilize their multifaceted functions. Concurrently, chondrocytes may transform or de-differentiate into fibroblast-like phenotypes, leading to the formation of fibrocartilage with suboptimal mechanical properties. This phenomenon indicates that inflammation is a significant barrier to effective cartilage repair in OA. Consequently, a comprehensive management strategy addressing the spatial and temporal aspects of joint inflammation is essential to foster an environment conducive to cartilage repair [21]. Hydrogels, known for their promising applications in tissue regeneration, offer a viable solution [220]. Their excellent biocompatibility and biodegradability make them suitable as cell delivery vehicles, providing physical protection to encapsulated cells from direct exposure to harsh microenvironments and aiding in the maintenance of cell viability [221]. Furthermore, hydrogels that mimic the natural ECM can enhance cell adhesion, spreading, and proliferation. Through physicochemical modifications, hydrogels can interact with immune cells and cytokines in the inflamed joint microenvironment. This interaction creates a favorable immune milieu for drugs or stem cells action, which benefits OA therapy.

Paracrine secretion is a crucial mechanism by which MSCs facilitate tissue regeneration [222]. However, an unsuitable adhesion microenvironment can impede this paracrine function. To resolve the limitation, *Li* et al. developed GelMA hydrogel microspheres (GMs) by combining platelet-derived growth factor-BB (PDGF-BB) with exogenous MSCs. The porous structure and excellent mechanical properties of GMs enhance the paracrine effect, allowing exogenous stem cells to adhere and proliferate on them [182]. The sustained release of PDGF-BB from GMs recruits endogenous MSCs and extends their paracrine activity. These GMs exhibit superior secretory properties and anti-inflammatory efficacy, slowing OA progression by optimizing the adhesion microenvironment and leveraging the synergistic effects of exogenous and endogenous MSCs. Additionally, sodium tanshinone IIA sulfonate (STS) can induce macrophage polarization from M1 to M2, inhibit apoptosis, and reduce inflammatory cytokine expression. The combined effect of these actions creates an immune environment favorable for regeneration, prevent articular cartilage degradation, and protect meniscal fibrochondrocytes [223,224]. *Li* et al. further advanced this approach by preparing an STS-loaded polycaprolactone (PCL)-meniscus extracellular matrix (MECM) hydrogel composite scaffold. The MECM-based hydrogel, with its excellent permeability to nutrients and metabolites, establishes an ideal microenvironment for cell adhesion, migration, proliferation, and differentiation [225].

### 4.3 Smart response Hydrogel

Stimulus-responsive hydrogels, also known as smart hydrogels, show great potential in various medical applications. They can respond to dynamic physiological and pathological environments by changing physical and chemical properties like temperature, pH, and chemical composition [226,227]. Stimulus-responsive hydrogels are particularly advantageous in the field of medicine because they are environmentally friendly, biocompatible, and can be tailored to specific environmental conditions. Their versatility has led to widespread use in drug delivery, biosensors, and tissue engineering [228]. The integration of smart and biomimetic 3D and 4D printing technologies has broadened the scope of hydrogel applications, including tissue engineering, cancer therapy, wound dressing, and the controlled release of bioactive agents and drugs [229]. Smart injectable hydrogels, in particular, are designed to respond rapidly to external stimuli such as light, temperature, pH, ion concentration, shear force, electric or magnetic fields, and enzymes. Such responsiveness facilitates the sustained and controlled release of therapeutics embedded within the hydrogels [230]. Consequently, smart injectable hydrogels serve as efficient and controllable platforms for local drug delivery, with the capacity to load and concentrate multiple immunotherapeutic agents [231]. In the treatment of OA, stimulus-responsive hydrogels can be injected into cartilage defect areas, where they form a gel-like substance with minimal invasiveness, providing structural support to damaged cartilage and promoting its repair process [232].

#### 4.3.1 Temperature-responsive hydrogels

Temperature-responsive hydrogels are a specialized class of hydrogels that undergo phase transitions in response to temperature fluctuations. Temperature-responsive hydrogels are predominantly composed of poly(N-isopropylacrylamide) (PNIPAAm) and its copolymers, which exhibit a low critical solubilization temperature (LCST) in water [233]. The LCST is the specific temperature at which temperature-responsive hydrogels experience a reversible phase transition, resulting in alterations in their swelling behavior and other properties [234]. Due to their unique ability to respond to external stimuli such as temperature changes, temperature-responsive hydrogels have garnered significant attention in various biomedical applications, particularly as controlled drug release systems [235]. Temperature-responsive hydrogels can be engineered to respond to specific temperature changes, enabling a targeted and controlled drug-release mechanism [236]. The capability of slow-release and localized drug delivery allows for high local concentrations of drugs, minimizing systemic toxicity and reducing the frequency of administration [237]. Furthermore, temperature-responsive hydrogels can be tailored to modulate the immune system, which is crucial in managing conditions like OA.

In the treatment of OA, temperature-responsive hydrogels can be utilized to deliver drugs that modulate the immune response associated with the disease. By integrating anti-inflammatory drugs into the hydrogel matrices, the release of these drugs can be managed based on the hydrogel's temperature-responsive properties [236]. This targeted drug delivery strategy effectively reduces inflammation and pain associated with OA by delivering drugs directly to the affected area in a controlled manner.

Thermo-responsive hydrogels owe their unique properties primarily to the temperature-responsive polymers they contain. Among these, the amphiphilic copolymer family known as poloxamers, or Pluronics, is particularly noteworthy due to its distinctive structural and property characteristics. Upon injection into the body, these polymers undergo a sol-gel transition, facilitating the long-term sustained release of drugs, which makes them essential materials for thermo-responsive hydrogels. For instance, *Chen* et al. utilized Pluronic F127 (PF127) combined with HA to load the small molecule △UA-diSE, resulting in a thermoresponsive sustained-release hydrogel. This hydrogel forms at body temperature and effectively enables the slow release of △UA-diSE, thereby significantly enhancing joint lubrication and improving anti-inflammatory effects within the cartilage microenvironment [238]. Similarly, García-*Couce* et al. developed injectable thermosensitive hydrogels by physically mixing CS with Pluronic-F127 (PF) and using tripolyphosphate (TPP) as a cross-linking agent. This formulation allows for the controlled release of dexamethasone, enhancing its anti-inflammatory efficacy [239]. Furthermore, *Zhang* et al. created thermosensitive hydrogels containing glucosamine (GlcN) based on poloxamer 407 and poloxamer 188, which form at body temperature (37 °C) [237]. These hydrogels maintain the release of glucosamine, effectively controlling inflammation and promoting cartilage regeneration. IA injection is a widely used therapeutic strategy for OA, aiming to control cartilage degradation and restore synovial fluid viscosity. To fully utilize the advantages of IA injection. *Diaz-Rodriguez* et al. combined heat-sensitive polymers (poloxamers) with HA to develop beta-lapachone (βLap)-loaded IA formulations. βLap, a naturally occurring naphthoquinone, has been recognized for its anti-inflammatory and wound-healing properties, making it a promising alternative therapeutic for OA [240]. Additionally, this formulation exhibits excellent rheological properties and significantly reduces the secretion of degradative molecules (MMP13) and pro-inflammatory molecules (CXCL8).

#### 4.3.2 Inflammation-responsive hydrogels

Inflammation-responsive hydrogels represent a cutting-edge class of biomaterials that have garnered considerable interest for their potential in treating various conditions, including OA, through immune system modulation. Inflammation-responsive hydrogels are engineered to respond to inflammatory signals *in vivo*, facilitating targeted drug delivery and immune response modulation [232]. They are characterized by their biocompatibility, design flexibility, and capacity to degrade while simultaneously releasing therapeutic agents in inflammatory environments [241]. Research has demonstrated that inflammation-responsive hydrogels can be employed for on-demand immunomodulation, such as expediting wound healing via epigenetic regulation of macrophages [242]. In the context of OA therapy, inflammation-responsive hydrogels can incorporate anti-inflammatory agents to inhibit inflammation at injury sites, thereby promoting healing and mitigating the inflammatory response [241]. Furthermore, their immunomodulatory properties are beneficial in modulating immune responses in scenarios like articular cartilage repair [243].

Among the various types of inflammation-responsive hydrogels, those sensitive to ROS and pH have shown particularly promising results [244]. For instance, *Yu* et al. developed a cartilage-targeted hydrogel microsphere with ROS responsiveness [245]. These microspheres optimize residence time in the joint cavity and enhance cellular uptake of therapeutic NPs. Upon encountering OA-induced intracellular ROS, the NPs depolymerize to neutralize excess ROS, reduce inflammation, and release DEX and chondroitin at the lesion site, effectively treating OA. Additionally, the hydrogel microspheres modulate the inflammatory response and promote chondrocyte differentiation, thereby slowing OA progression by intervening in anti-inflammatory and pro-inflammatory pathways and facilitating cartilage repair. Moreover, mitochondrial dysfunction and excessive ROS accumulation create a vicious cycle of increased senescent cells [246]. In stem cell therapy, microvesicles (MVs) released by MSCs, ranging from 100 nm to 1 μm in diameter, are pivotal for therapeutic success [247]. These MVs are rich in bioactive molecules, including miRNAs, proteins, and cytokines, which regulate tissue physiology and metabolism, attenuate oxidative stress, stimulate tissue regeneration, and ameliorate cellular senescence by modulating mitochondrial energy metabolism [248]. To enhance the immunomodulatory activity and content of MVs under varying stimulation conditions, *Liu* et al. utilized IFN-γ as a specific stimulus to generate IFN-γ-microvesicles with enhanced anti-aging effects. They also prepared 3-aminophenylboronic acid-modified silk fibroin (SF) and PVA as ROS-responsive vectors to protect MVs, prolong their activity, and facilitate smart release [249].

**Fig. 5:**
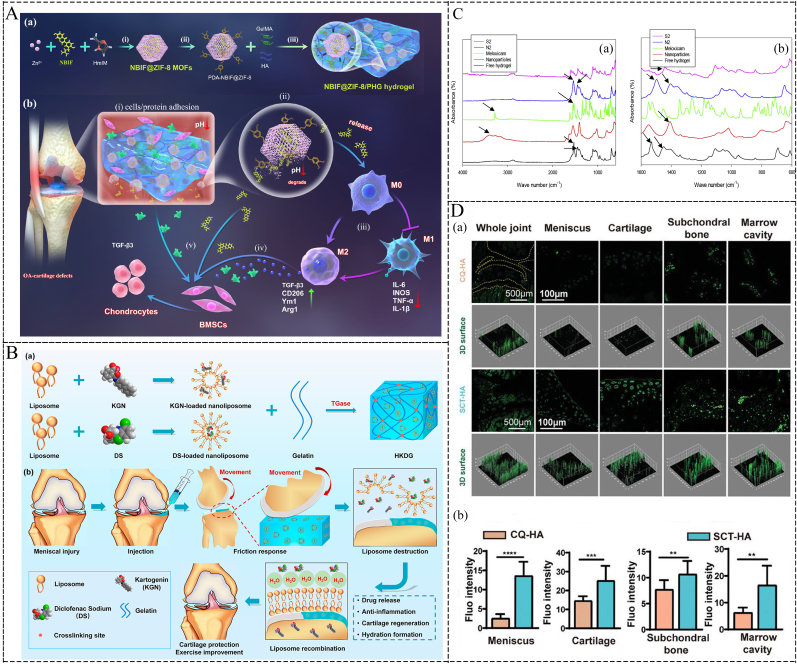
**Immunomodulation-based hydrogels for OA therapy.** (A) Schematic diagram of hydrogel design and application for promoting OA-induced cartilage defect repair. Copyright 2023, American Chemical Society [251]. (B) Schematic Illustration of Preparation and Application of the Hydrogel for Treatment of Cartilage Degradation Induced by Meniscal Tears. Copyright 2023 American Chemical Society [252]. (C) ATR-FTIR spectra of the free hydrogel, meloxicam loaded nanoparticles, meloxicam, N2 and S2 hydrogels (a) the scanning range 4000-600 cm^−1^, and (b) the enlarged overlay range 1600-600 cm^−1^. Copyright 2019, Elsevier [253]. (D) SB permeation of SCT-HA *in vivo*. (a) Whole joint, meniscus, cartilage, SB, and bone marrow cavities of mouse knee joint sections were visualized by laser scanning confocal observation and 3D surface reconstruction after CQ-HA and SCT-HA were injected into the joint cavity. (b) Fluorescence intensity analysis of CQ-HA and SCT-HA penetrating the meniscus, cartilage, SB, and bone marrow cavities of mouse knee joints. Copyright 2024, John Wiley and Sons [254].

In OA-injured joints, the synovial fluid pH decreases from 7.4 to 6.0 due to local tissue hypoxia metabolism and acidosis, making this significant pH change an ideal condition for modulating drug delivery. *Jiang* et al. developed a hydrogel that responds to the inflammatory microenvironment of joints by integrating a MOF to control the release of anti-inflammatory compounds, specifically neobavaisoflavone (NBIF) [250]. NBIF was encapsulated in zeolite imidazole frameworks (ZIF-8 MOFs) and integrated into a gelatin methacrylate/HA hybridization network to form an NBIF@ZIF-8/PHG hydrogel. NBIF@ZIF-8/PHG hydrogel releases NBIF under weakly acidic conditions in damaged joints, thereby reducing inflammation and promoting the differentiation of stem cells into chondrocytes, contributing to cartilage repair. Additionally, NBIF aids in managing the inflammatory environment and healing cartilage defects by modulating the immune environment and inhibiting key inflammatory factors such as IL-1β, IL-6, and TNF-α, highlighting the potential of immunomodulation in treating OA (Fig. 5A).

#### 4.3.3 Mechanical friction-responsive hydrogels

Mechanically responsive hydrogels are highly valued for their adaptability to external stimuli, making them versatile materials for a wide range of applications. In the treatment of OA through immunomodulation, mechanically responsive hydrogels present promising opportunities for therapeutic intervention. By incorporating immunomodulators into mechanoresponsive hydrogels, a system can be developed that responds to mechanical cues in the joint environment, thereby modulating the immune response and reducing inflammation associated with OA [255]. The application of mechanically responsive hydrogels in OA therapy necessitates the design of materials with specific mechanical properties that can endure the mechanical stresses of the joint while also providing sustained drug release capabilities. Inspired by the composition and function of menisci, *Liu* et al. developed an injectable hydrogel encapsulating nanoliposomes with self-lubricating and friction-responsive properties. This innovative design leverages the frictional force sensitivity of the nanoliposomes, which are released from the hydrogel when subjected to friction within the joint. Upon release, the nanoliposomes reassemble on the hydrogel's surface to form a hydrated layer, aiding in joint lubrication and friction reduction [256]. As the nanoliposomes are released, they gradually release encapsulated DS and KGN into the joint cavity, providing anti-inflammatory effects and promoting cartilage regeneration (Fig. 5B).

### 4.4 Nanocomposite hydrogels

Nanomaterial composite hydrogels represent an innovative class of biomaterials that integrate nanomaterials into a 3D hydrogel network through physical or chemical covalent interactions. This integration effectively mitigates the limitations associated with using nanomaterials or hydrogels individually, thereby offering significant potential in the biomedical field. Nanomaterials are renowned for their nanoscale size, diverse structures, and exceptional mechanical, electrical, thermal, and photoresponsive properties, making them highly promising across various applications [257]. However, their small size can lead to challenges such as sudden release or rapid clearance by the reticuloendothelial system, as well as poor bioadhesion, which may result in off-target effects [258]. Conversely, hydrogels are extensively utilized in drug and cell delivery, tissue repair scaffolding, ECM substitution, and artificial organ mimicry [259,260]. Despite their versatility, hydrogels often suffer from weak mechanical properties and limited reactivity, which constrain their practical applications [261].

In nanocomposite hydrogel systems, multifunctional nanomaterials frequently serve as a functional core, imparting a range of properties such as photothermal and magnetothermal conversion, electrical conductivity, and targeting capabilities to the composite system [[262], [263], [264]]. While hydrogels are typically more suited for delivering hydrophilic drugs, nanocomposite hydrogels excel in delivering hydrophobic drugs. For instance, *Seo* et al. explored the localized and sustained delivery of triamcinolone acetate (TCA) using polymeric nanoparticle (PN) hydrogel systems based on poly(organophosphazenes) [265]. Hydrophobic drugs can be effectively encapsulated into PNs through self-assembly, driven primarily by hydrophobic interactions between side chains [266]. During this process, water-insoluble TCA is readily incorporated into the hydrophobic core of the PN, forming TCA-encapsulated PN (TePN) particles. The resulting 3D TePN hydrogel facilitates the prolonged release of TCA over several months, which inhibits MMP expression in cartilage by modulating cytokine expression, thereby treating OA. The integration of nanostructures within hydrogels also enhances their mechanical properties. For example, *Fattahpour* et al. developed an injectable hydrogel composed of carboxymethyl chitosan (CMC), methylcellulose (MC), pluronic (P), and zinc chloride, incorporating meloxicam. Despite the inherent advantages of CMC scaffolds, they exhibit poor mechanical properties, which were addressed by reinforcing them with MC, dispersing them with P, and crosslinking with zinc ions [253] (Fig. 5C). Hydrogels can significantly improve the retention of nanomaterials, providing the composite system with the plasticity required for diverse biomedical applications. Furthermore, the design of nanocomposite hydrogels can be tailored to specific conditions, such as joint lubrication for OA. *Chen* et al. developed an injectable nanocomposite hydrogel comprising polygallocatechin-manganese (PGA-Mn) NPs, oxidized sodium alginate, and gelatin [267]. The incorporation of PGA-Mn not only ensured effective ROS scavenging but also enhanced the mechanical strength of the biohydrogel through a Schiff base reaction with gelatin. Additionally, the hygroscopic properties of the hydrogel reduce intra-articular friction, promote the production of cartilage-associated proteins, and support cartilage synthesis. Notably, certain nanocomposite hydrogels possess intrinsic bioactivities that enable them to actively participate in mediating the immune microenvironment within osteoarthritic joints. Furthermore, nanocomposite hydrogels enhance the efficiency of drug delivery, thereby contributing to improved therapeutic outcomes. In a study by *Zuo* et al., ultra-small selenium-doped carbon quantum dots (Se-CQDs, approximately 5 nm in size) were synthesized using a hydrothermal method. These Se-CQDs were then coupled with triphenylphosphine (TPP) to create TPP-Se-CQDs (SCT). Subsequently, SCT was dynamically complexed with acetaldehyde- and methacrylato anhydride-modified hyaluronic acid (AHAMA). The construction of highly permeable micro/nanohydrogel microspheres (SCT@AHAMA) has been demonstrated to enhance the effective penetration of articular subchondral bone, as illustrated in Fig. 5D [254] (Fig. 5D).

## 5 Scaffold

In the context of OA, excessive release of inflammatory cytokines impairs cartilage repair mechanisms. Abnormal proliferation and sclerosis of subchondral bone contribute to joint space narrowing, osteophyte formation, and pathological bone remodeling [268]. Such osteochondral defects result in joint instability and loss of function; without timely intervention, these issues will exacerbate OA progression. Osteochondral defects cause joint instability and loss of function. Without timely intervention, this instability and dysfunction accelerate OA progression [269]. Scaffolds provide mechanical support to stabilize damaged areas and prevent further injury. They also possess a highly porous structure with appropriate pore sizes, promoting cellular attachment, migration, and proliferation [270]. The selection of scaffold materials and surface modification further promote the adhesion and differentiation of chondrocytes and MSCs, thus enhancing cartilage and bone tissue regeneration [270].

**Fig. 6:**
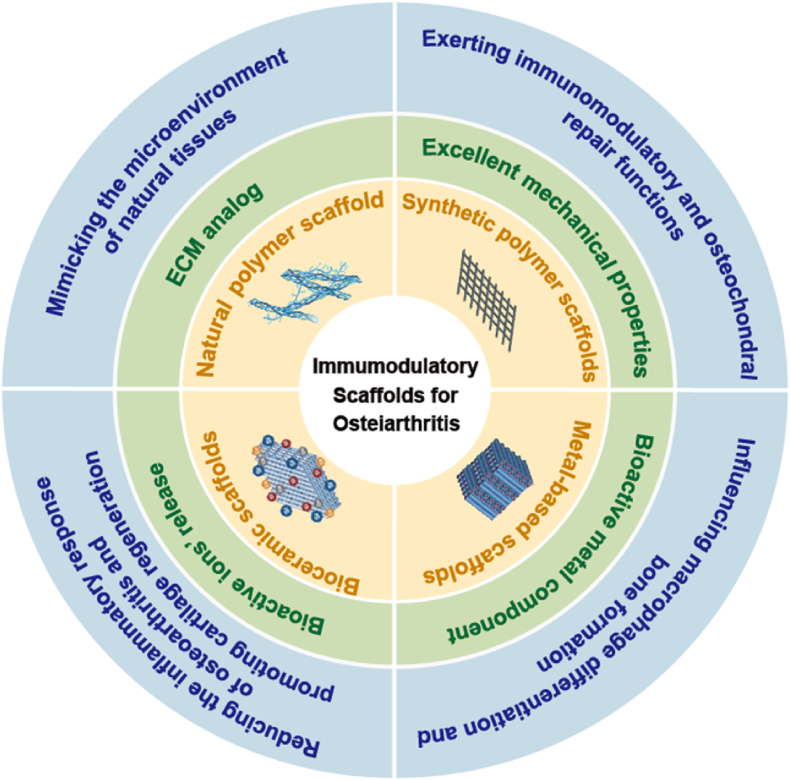
**The classification, utilization and therapeutic effects of immumodulatory scaffolds for OA.** The immumodulatory scaffolds are divided into four groups, including natural polymer scaffolds, synthetic polymer scaffolds, bioceramic scaffolds and metal-based scaffolds. They play anti-inflammatory and cartilage repair functions through the properties described in this picture to promote OA prognosis.

However, implanted scaffolds often trigger an inflammatory response. The implantation of scaffolds activates various biochemical signals associated with inflammation, leading to the recruitment of different types of immune cells at the implantation site. Upon recruitment, monocytes differentiate into macrophages under the influence of cytokines synthesized and secreted by other immune cells. Subsequently, the differentiated macrophages begin to adhere to the surface of the implant. The plasticity of these adherent macrophages is largely controlled by the physicochemical properties of the implanted scaffold, significantly impacting the bone regeneration process [271]. Therefore, advanced scaffold technologies must consider material biocompatibility and mechanical properties. They also require excellent immunomodulatory capabilities to reduce inflammation and promote simultaneous cartilage and bone regeneration in inflamed joints. This section examines the use of natural polymer, synthetic polymer, and composite biomaterial scaffolds for OA treatment. Composite scaffolds discussed include bioceramic, metal-based, and other specialized types (Fig. 6). Key applications include modulating immune reactions and promoting osteochondral repair.

### 5.1 Natural polymer scaffolds

Natural polymers are highly attractive biomaterials in the field of tissue engineering because they closely resemble the components of the ECM in the body, thereby more effectively mimicking the microenvironment of natural tissues [270]. Sourced from plants, animals, and microorganisms, natural polymers encompass a variety of materials such as collagen, cellulose, alginate, CS, and silk fibroin. Consequently, compared to synthetic materials, natural polymer scaffolds exhibit low toxicity, low immunogenicity, excellent biocompatibility, and robust capabilities in promoting cell growth and adhesion [272]. Natural polymer scaffolds offer significant therapeutic potential for OA. Their ability to modulate inflammatory microenvironments enables dual functionality in immunoregulation and osteochondral regeneration.

Collagen scaffolds, in particular, play a crucial role in bone tissue engineering as they can closely mimic the natural bone structure [273]. *Court* et al. demonstrated that 3D type I collagen scaffolds can influence the polarization of M2 macrophages by regulating Signal Transducer and Activator of Transcription 1 phosphorylation via integrin β2, thereby affecting macrophage responses to IFN-γ/LPS [274]. Notably, compared to mammalian collagen scaffolds, jellyfish collagen scaffolds can induce a more pronounced anti-inflammatory tissue response [275]. As a result, collagen-derived scaffolds are widely fabricated and utilized. *Bauza* et al. designed a biomimetic collagen-chondroitin sulfate scaffold that promoted the expression of anti-inflammatory cytokines such as Adiponectin, IL1RA, and IL10, while inhibiting the expression of pro-inflammatory cytokines like IL23A, Chemokine (C‐X3‐C motif) ligand 1, Bone morphogenetic protein 2, and Oncostatin M‐like. Furthermore, the scaffold enhanced the expression of cartilage-specific genes such as *ACAN* and *COL2A1*, achieving dual functions in cartilage repair and inflammation regulation [276]. Additionally, it is noteworthy that the implantation of Col2 scaffolds under surgically induced OA conditions can repair critical-sized osteochondral defects by inhibiting the TGF-β-Smad1/5/8 signaling pathway [277].

Among polysaccharides, CS and its derivatives are ideal materials for bone tissue engineering due to their low toxicity, excellent biocompatibility, biodegradability, and various bioactivities, including antibacterial, antioxidant, and antitumor properties [278]. Molecular weight modifications and functionalization strategies, such as carboxymethylation or nanoparticle incorporation, further enhance their immunomodulatory potential [279]. *Patel* et al. incorporated SF and cellulose nanoparticles (CNP) into CS and used them as enhancers for bone tissue regeneration to prepare CS/SF/CNP composite scaffolds. This scaffold can exert bone immunomodulatory effects and promote the polarization of macrophages to the M2 phenotype, thereby facilitating bone tissue regeneration [280]. Additionally, *Tang* et al. surface-modified CS by attaching specific peptide sequences (SDSSD) to the scaffold (CS-SDSSD), simulating the development process of bone tissue from the fibrous membrane [281]. Studies have shown that CS-SDSSD scaffolds can specifically bind to the periosteum via the peptide sequence, attract and organize the migration of osteoblasts, and promote the polarization of M2 macrophages, accelerating the repair process of subperiosteal ossification [281].

**Fig. 7:**
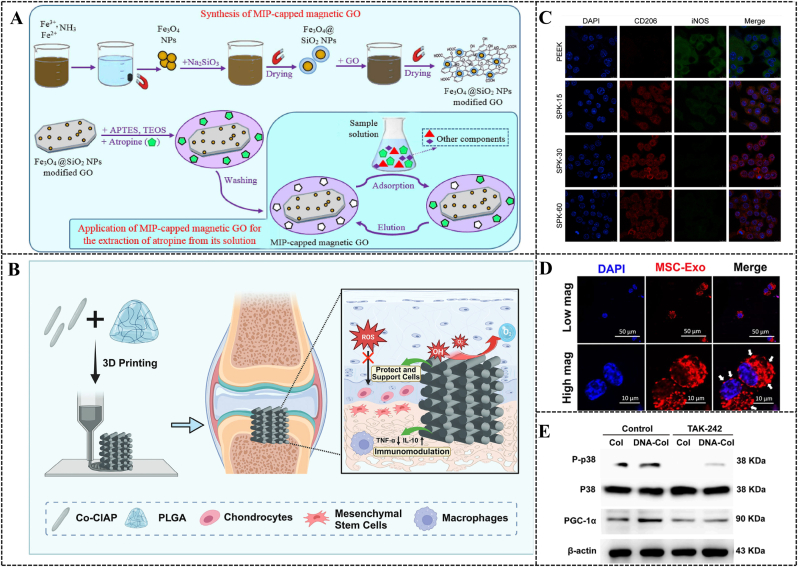
**Immunomodulation-based scaffolds for OA therapy.** (A) Schematic procedure for the synthesis of MIP-modified magnetic GO and its application for the cyclic extraction of atropine. Copyright 2019, Elsevier [285]. (B) Schematic illustration of 3D-printed Co-ClAP/PLGA scaffolds for the treatment of osteochondral defects containing excess ROS. Copyright 2023, John Wiley and Sons [286]. (C) Scaffolds with porous sulfonated PEEK scaffolds for macrophage polarization modulation: immunofluorescence staining of macrophages (RAW264.7) cultured with PEEK, SPK-15, SPK-30, and SPK-60 for 4 days. Copyright 2023, Elsevier [287]. (D) Confocal microscopy imaging of internalized releases from PLA-Exo scaffolds into pro-inflammatory macrophages. Exo. Cells were stained with DAPI (blue) and examined by confocal laser scanning microscopy. Copyright 2023, EMC [288]. (E) Mechanisms by which DNA-Col promotes Treg differentiation through the TLR4-p38-PGC-1α pathway. By using the TLR4 inhibitor TAK-242, the investigators found that the promotion of PGC-1α expression and p38 phosphorylation by DNA-Col was attenuated, suggesting that DNA-Col may regulate T-cell metabolism and differentiation by activating TLR4. Copyright 2023, Elsevier [284] (For interpretation of the references to colour in this figure legend, the reader is referred to the Web version of this article.)

Nucleic acids, as a special class of natural polymers, have been widely explored for their immunological properties [282]. For example, immunoregulatory RNA molecules (imRNA) can stabilize calcium phosphate ion clusters to produce imRNA-ACP, and collagen scaffolds containing imRNA-ACP can activate the JAK2-STAT3 signaling pathway in macrophages. Activation of this pathway leads to the polarization of macrophages to the M2 phenotype, thereby regulating the bone immune microenvironment to promote bone regeneration [283]. Besides RNA, *Song* et al. designed a DNA-conjugated collagen scaffold, and experiments showed that DNA-Col scaffolds could promote T-cell differentiation by activating the TLR4-p38-PGC-1 α pathway. Moreover, DNA-Col scaffolds can provide an anti-inflammatory environment to support osteogenesis by promoting T-cells to secrete anti-inflammatory cytokines such as IL-4 and IL-10 [284] (Fig. 7E).

### 5.2 Synthetic polymer scaffolds

Although natural polymers support cell adhesion and function, they often exhibit poor mechanical properties and limited availability [289]. Therefore, synthetic polymers have gained significant popularity. Polymers such as PLA, poly(hydroxybutyrate-co-3-hydroxyvalerate), polyglycolic acid, PCL, poly-3-hydroxybutyrate, poly-ether-ether-ketone (PEEK), PLLA, and poly(lactic-co-glycolic acid) (PLGA) are widely used in bone tissue engineering [290]. Many of these materials can exert immunomodulatory and osteochondral repair functions to treat OA [291]. In terms of immunomodulation, high-precision fabrication methods can control the porosity, pore size, and geometric shape of synthetic polymers. For instance, *Jiang* et al. modified PCL using digital light processing 3D printing technology to produce methacrylated polycaprolactone scaffolds suitable for high-precision photopolymerization printing. They used biotin-streptavidin as a connecting molecule to effectively and firmly bind engineered vesicles to the scaffold [292]. Early immunofluorescence staining results showed a decrease in M1 macrophages and an increase in M2 macrophages in the tissue surrounding the implanted biomimetic osteoinductive scaffold, which is conducive to improving the early microenvironment at bone defect sites [292].

Since synthetic polymers are easier to functionalize compared to natural polymers, surface modification of synthetic polymer scaffolds to achieve dual functions of immunomodulation and bone tissue repair and regeneration is also a promising strategy [293]. For example, high-performance PEEK has garnered significant attention in the medical field due to its exceptional strength, making it suitable for high-load applications [294]. *Yuan* et al. 3D-printed a PEEK scaffold and sulfonated its surface. Compared to untreated PEEK scaffolds, sulfonated PEEK scaffolds could induce macrophages to polarize to the M2 phenotype, increase the secretion of anti-inflammatory cytokines such as IL-4 and IL-10, and reduce the production of pro-inflammatory cytokines such as TNF- α and IL-1β. This helps alleviate the local inflammatory environment and protect cartilage from damage [287] (Fig. 7C). Similarly, *Shi* et al. applied a calcium phosphate (CaP) coating to the surface of PCL scaffolds. Compared to bare PCL scaffolds, CaP-coated PCL scaffolds exhibited a rougher surface morphology and higher hydrophilicity. The surface morphology of the coating and the release of calcium ions were crucial in regulating the transition of macrophages from the M1 to the M2 phenotype. They may activate the Phosphoinositide 3-Kinase/Protein Kinase B and cyclic adenosine monophosphate- Protein Kinase A pathways, respectively, to promote M2 polarization [295]. *Zhang* et al. 3D-printed PLA scaffolds, then isolated MSC-derived Exo from human bone marrow-derived MSCs and attached the Exo coating to the PLA scaffold surface. Experimental results indicated that PLA-Exo scaffolds could reduce the expression of pro-inflammatory markers and the production of ROS, demonstrating potential immunomodulatory capabilities. This helps mitigate excessive inflammatory responses and promote tissue repair [288] (Fig. 7D).

### 5.3 Bioceramic composite scaffolds

Bioceramics themselves not only exhibit activities that promote cell proliferation, induce angiogenesis, and provide antimicrobial surfaces, but also release bioactive ions such as calcium, magnesium, and silicon upon degradation. Bioceramics scaffolds can reduce the inflammatory response of osteoarthritis and promote cartilage regeneration by regulating macrophage polarization [296], balancing the release of inflammatory factors [297], regulating T cell differentiation [298], stimulating the regeneration of chondrocytes and osteoblasts, and anti-oxidative stress [299]. These bioactive ions can effectively regulate stem cell functions and promote tissue remodeling [300]. Additionally, silicate bioactive glass represented by 45S5 bioactive glass and calcium phosphate ceramics such as β-tricalcium phosphate have been shown to regulate macrophage polarization through *in situ* mineralization, triggering "calcium oscillations" in macrophages [301]. Recently, the biphasic calcium phosphate and mesoporous bioactive glass have also been explored for their roles in regulating macrophage polarization [302,303]. Bioceramic scaffolds can be further modulated to enhance immunoregulation. Physical modifications—including surface architecture, pore size, and porosity—optimize structure. Chemical modifications like surface charge adjustment and metal ion incorporation also boost immunoregulatory potential [304].

#### 5.3.1 Bioceramic-polymer scaffolds

Bioceramics are widely used in bone tissue engineering, but their inherent high brittleness and low ductility result in poor processability, limiting their clinical applications [305]. However, combining bioceramics with polymers can significantly improve the mechanical properties of the scaffolds. *Hu* et al. utilized hydrogen bonding interactions between PVA and SF, as well as possible ionic interactions between SF and ceramic particles, allowing the scaffold to fully recover its original shape under 35 % compressive strain, thereby enhancing its toughness [305]. *Del-Mazo-Barbara* et al. developed a novel self-hardening ink 1containing active ceramic particle in a PCL solution, replacing traditional hydrogel binders. This method successfully improved the mechanical strength and toughness of the scaffold while maintaining over 90 wt% bioactive ceramic phase [306]. These studies indicate that polymer-ceramic scaffolds not only retain the immunoregulatory effects of bioceramics but also effectively address brittleness limitations. Furthermore, various modifications can be applied to polymer-ceramic scaffolds to exploit their immunoregulatory potential, such as altering surface structures, pore sizes, porosity, and surface charges [304]. For example, to address the hydrophobicity issue of PCL/hydroxyapatite scaffolds, *Li* et al. treated the scaffolds with different concentrations of sodium hydroxide. They identified 2.0 and 2.5 mol/L as optimal concentrations that exhibited good hydrophilicity and mechanical properties. These treated scaffolds successfully regulated macrophage polarization [307]. Additionally, *Li* et al. found that PCL/PEG/Hydroxyapatite scaffolds with 600-μm pore size outperformed those with 200-μm and 400-μm pore sizes in reducing foreign body reactions, promoting M2 macrophage infiltration, vascular growth, and new bone formation [308].

#### 5.3.2 Bioceramic-nanomaterial composite scaffolds

Bioceramic-nanomaterial composite scaffolds are solid polymer composites embedded with nanoscale materials, where at least one component has a size range of 1–100 nm [309]. The addition of nanomaterials to biopolymer composites can improve their properties [310]. Given the pathological characteristics of OA, the inhibitory effects of nanomaterials on the inflammatory microenvironment are particularly noteworthy. For example, NP-based therapies have shown potential in treating severe inflammation [311]. *Deng* et al. combined 3D-printed calcium hydroxyapatite with hair-derived antioxidant nanoparticles (HNPs) and microparticles (HMPs) to develop a unique bioceramic scaffold. The HNPs and HMPs in the scaffold mimic the activity of SOD and CAT, scavenging superoxide anions and hydrogen peroxide, thereby reducing damage to chondrocytes in the inflammatory environment [299]. Besides incorporating NPs, nanomaterials can also be doped or added in nanofiber forms. *Zhang* et al. used MnCO nanosheets to generate CO and Mn^2+^, promoting macrophage polarization to the M2 phenotype [312]. *Liu* et al. combined electrospun PLLA (PLA) microfibers and nanofibers with 3D-printed PCL scaffolds, promoting RAW264.7 cells to polarize more towards the alternatively activated macrophage (M2 type) [291]. Additionally, nanocarbon-based materials such as graphene oxide (GO) have been found to induce immunomodulatory responses that affect macrophage phenotypes [313]. *Qi* et al. designed a scaffold primarily composed of 3D-printed porous bioactive glass and GO. The rough hydrophilic surface of GO led to the upregulation of surface-dependent WNT-3A protein by enhancing integrin signaling and the accumulation and activation of intracellular β-catenin protein. Activation of this signaling pathway is crucial for macrophage polarization and osteogenic differentiation [314].

#### 5.3.3 Bioceramic-metal ion composite scaffolds

Various metal ions have demonstrated excellent anti-inflammatory capabilities. For example, zinc/cobalt bimetallic MOF exhibits outstanding ROS catalytic activity, similar to peroxidase (POD), CAT, and SOD [285] (Fig. 7A). Magnesium ions have also been shown to regulate macrophage polarization states [315]. Therefore, metal-doped scaffolds have immunomodulatory potential in OA, capable of inflammation inhibition and osteochondral repair. *Shu* et al. prepared various cobalt-containing scaffolds, demonstrating their ability to scavenge ROS while also repairing cartilage and subchondral bone [316,286] (Fig. 7B). Additionally, *Lin* et al. developed copper-incorporated bioactive glass ceramic scaffolds (Cu-BGC). Cu-BGC scaffolds were found to induce macrophages to transition from the M0 (unactivated) state to the M2 phenotype. Cu-BGC reduced the expression of pro-inflammatory cytokines (e.g., TNF-α, IL-18) and increased the expression of anti-inflammatory cytokines (e.g., IL-10). This transition helps to mitigate the inflammatory response caused by OA and slows down the damage to cartilage and subchondral bone [297].

In addition to single metal incorporation, MOFs can be constructed by integrating specific metal cations or clusters with organic ligands to create catalytic active sites. MOFs with broad-spectrum ROS-scavenging capabilities can be prepared, and as the MOF degrades, it releases various biologically active metal ions [316].

### 5.4 Metal-based scaffolds

Metals can also be used to fabricate functionalized scaffolds for immunomodulation, with titanium-based scaffolds being the most typical example. Smooth titanium induces the activation of M1-like inflammatory macrophages by increasing the levels of proteins such as IL-1β, IL-6, and TNF-α. In contrast, rough titanium promotes anti-inflammatory M2 macrophages that support tissue regeneration [317]. Adjustments to the physical structure, such as endowing the surface with hydrophilic properties and modifying the porosity and pore size of the titanium-based scaffolds, can regulate macrophages [318]. *Bai* et al. chemically modified the titanium scaffold surface to create biomimetic peptide-coated scaffolds. Using the strong coordination interaction between catechol groups in the peptide and surface titanium oxide, as well as the regulatory and osteogenic effects of the biomimetic peptide coating on macrophages, they promoted bone regeneration in OA patients [170].

In addition to physical and chemical properties, the composition of the metal can also influence macrophage polarization, potentially affecting osteogenesis. Titanium-based materials containing silver NPs significantly increased the expression of CD206 (a marker of M2 macrophages) and decreased the expression of iNOS (a marker of M1 macrophages), promoting M2 macrophage polarization. This helps to reduce inflammation and induce osteogenesis in infected bone defects [319]. Titanium-copper alloy (Ti-5Cu) facilitated the transition of macrophages from the M1 type to the M2 type by lowering the expression of TNF- α and increasing the expression of IL-10, accelerating osteoblast differentiation and bone formation [320].

### 5.5 Special composite biomaterial scaffolds

Special composite biomaterial scaffolds have also been developed. *Gomes* et al. mixed choline-based caffeate (Ch [Caffeate]) a biocompatible ionic liquid based on phenolic compounds with other natural polymers to create bio-inks. Ch[Caffeate] can reduce the release of pro-inflammatory cytokines (e.g., TNF- α and IL-6) and mediators from THP-1 macrophages, which play crucial roles in the early stages and progression of OA [321].

## 6 Summary and Outlook

In this review, we have systematically examined the immune mechanisms that drive OA progression and explored how biomaterial innovations are being leveraged to modulate these pathways. The increasing recognition of the immune system's role in biomaterial integration has reshaped the development of therapeutic strategies, aligning material design with immunological principles to create more effective interventions.

Recent advancements in biomaterials have evolved beyond their traditional structural roles to actively modulate immune responses. By fine-tuning properties such as morphology, surface chemistry, and bioadhesion, these materials can regulate the inflammatory microenvironment, control immune cell recruitment, and facilitate cartilage and bone regeneration. Moreover, biomaterial scaffolds have emerged as platforms for the controlled release of bioactive agents, including cytokines, growth factors, and immunomodulatory drugs, enabling precise and sustained therapeutic effects.

Despite these advances, several challenges remain. The translation of immunomodulatory biomaterials into clinical applications requires deeper understanding of long-term biocompatibility, personalized immune responses, and the optimization of biomaterial degradation profiles. Future research should focus on refining biomaterial design to achieve more targeted immune regulation while ensuring safety and scalability for clinical use. Additionally, interdisciplinary collaborations between immunologists, material scientists, and clinicians will be crucial for accelerating the development of next-generation biomaterials that not only mitigate OA progression but also actively promote tissue repair.

By integrating immunomodulatory principles with biomaterial engineering, we are moving toward a new paradigm in OA therapy—one that harnesses the immune system's regenerative potential to restore joint function and improve patient outcomes.

## CRediT authorship contribution statement

**Ruizhe Zhao:** Writing – original draft, Visualization, Investigation, Conceptualization. **Bing Liang:** Writing – original draft. **Yijie Shi:** Writing – original draft, Visualization, Investigation, Conceptualization. **Jianfei Gao:** Writing – original draft, Visualization. **Xuezhe Wang:** Visualization. **Tianyi Shao:** Visualization. **Kunyue Xing:** Writing – review & editing. **Mingzhe Yan:** Validation, Supervision, Project administration, Conceptualization. **Tianrui Wang:** Supervision, Project administration. **Yingze Zhang:** Writing – review & editing, Project administration, Conceptualization. **Dongming Xing:** Writing – review & editing, Supervision, Project administration, Funding acquisition, Conceptualization.

## Ethics approval and consent to participate

This review article does not require any allied consent or ethical approval for publication.

## Declaration of competing interest

The authors declare that they have no known competing financial interests or personal relationships that could have appeared to influence the work reported in this paper.

## Data Availability

No data was used for the research described in the article.
